# Insights into Protein Aggregation by NMR Characterization of Insoluble SH3 Mutants Solubilized in Salt-Free Water

**DOI:** 10.1371/journal.pone.0007805

**Published:** 2009-11-23

**Authors:** Jingxian Liu, Jianxing Song

**Affiliations:** 1 Department of Biochemistry, Yong Loo Lin School of Medicine, National University of Singapore, Singapore, Singapore; 2 Department of Biological Sciences, Faculty of Science, National University of Singapore, Singapore, Singapore; Massachusetts Institute of Technology, United States of America

## Abstract

Protein aggregation *in vivo* has been extensively associated with a large spectrum of human diseases. On the other hand, mechanistic insights into protein aggregation *in vitro* were incomplete due to the inability in solubilizing insoluble proteins for high-resolution biophysical investigations. However, a new avenue may be opened up by our recent discovery that previously-thought insoluble proteins can in fact be solubilized in salt-free water. Here we use this approach to study the NMR structural and dynamic properties of an insoluble SH3 mutant with a naturally-occurring insertion of Val22 at the tip of the diverging turn. The obtained results reveal: 1) regardless of whether the residue is Val, Ala, Asp or Arg, the insertion will render the first hNck2 SH3 domain to be insoluble in buffers. Nevertheless, all four mutants could be solubilized in salt-free water and appear to be largely unfolded as evident from their CD and NMR HSQC spectra. 2) Comparison of the chemical shift deviations reveals that while in V22-SH3 the second helical region is similarly populated as in the wild-type SH3 at pH 2.0, the first helical region is largely unformed. 3) In V22-SH3, many non-native medium-range NOEs manifest to define non-native helical conformations. In the meanwhile a small group of native-like long-range NOEs still persists, indicating the existence of a rudimentary native-like tertiary topology. 4) Although overall, V22-SH3 has significantly increased backbone motions on the ps-ns time scale, some regions still own restricted backbone motions as revealed by analyzing ^15^N relaxation data. Our study not only leads to the establishment of the first high-resolution structural and dynamic picture for an insoluble protein, but also shed more light on the molecular events for the nonhierarchical folding mechanism. Furthermore, a general mechanism is also proposed for *in vivo* protein aggregation triggered by the genetic mutation and posttranslational modification.

## Introduction

Protein aggregation in cells is emerging as common features of the diseases, in particular for a large array of neurodegenerative diseases such as Alzheimer's disease (AD), Parkinson's disease (PD), Huntington's disease (HD), amyotrophic lateral sclerosis (ALS) and prion diseases. Also it has been shown that many of these diseases result from protein aggregation due to a genetic mutation and posttranslational modification in the sequence of the disease-related protein. Even for proteins that do not cause direct aggregation diseases, their aggregates may cause toxicity to cells [1]–[3]. As a consequence, an in-depth understanding of the biochemistry and biophysics of the aggregation processes will be crucial to unravelling fundamental mechanisms underlying these diseases as well as to further developing therapeutic strategies and agents.

Protein aggregation is a multi-step process and unfortunately most disease-causing protein mutants have been characterized to be highly insoluble even *in vitro*. Previously there was no general method to solubilize insoluble proteins for high-resolution biophysical investigations. Consequently conformational properties of the initial states of aggregation were unknown and the underlying mechanisms are largely incomplete. However, recently we discovered that the previously-thought insoluble proteins, one even with transmembrane fragment, could in fact be solubilized in salt-free water for detailed biophysical studies [4]–[11]. Now this approach has also been used by other groups to investigate aggregation-prone proteins [12]–[14].

**Figure 1:**
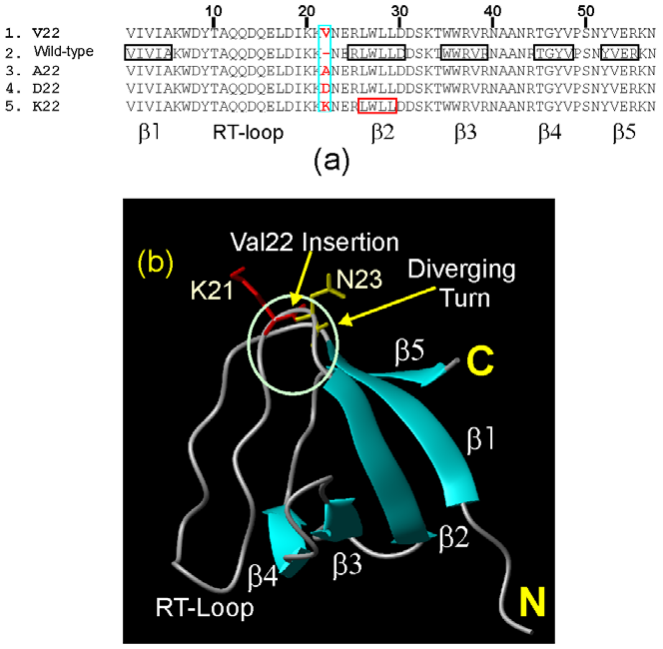
Sequence and structure of the first hNck2 SH3 mutants. (a). Sequence alignments of different hNck2 SH3 proteins with secondary structure regions boxed and labelled. 1. V22-SH3 previously derived from the hNck2 sequence AAC04831 with an extra Val insertion at position 22. 2. wild-type SH3. 3. A22-SH3 with the extra Val22 mutated to Ala. 4. D22-SH3 with the extra Val22 mutated to Asp. 5. K22-SH3 with the extra Val22 mutated to Lys. (b). The published NMR structure of the first hNck2 SH3 domain (2B86) with the secondary structures and position of the inserted Val residue indicated.

So far, more than 4,000 SH3 modular domains have been identified in a variety of organisms. The SH3 domains, containing ∼60 residues and no disulfide bridge, play a critical role in transmitting as well as integrating cellular signals [15]–[17]. Structurally, all SH3 domains share a common β-barrel fold comprising five β-stands, which are organized into two β-sheets. Very surprisingly, we recently reveal that the first human Nck2 (hNck2) SH3 domain adopting a classic SH3 fold in the native condition [18] suddenly becomes highly helical upon being destabilized by acid at pH 2.0 or 4-residue mutations on the second β-strand [11]. On the other hand, there are several hNck2 sequences deposited in GenBank. Originally we coincidently selected the hNck2 protein sequence with GenBank code of AAC04831 for deriving the sequence of its first hNck2 SH3 domain. Very surprisingly, we found our recombinant SH3 domain to be neither soluble nor refoldable in aqueous buffers [10] but had no explanation until its NMR structure was reported [8]. It turned out that our construct had an extra Val insertion at the position 22 of the first SH3 domain (Figure 1a), which is only found in the hNck2 sequence AAC04831 but not other such as AAC80284. Intriguingly, in this insertion mutant we designated as V22-SH3, the extra residue Val is located at the tip of the diverging turn linking the RT-loop and the second β-strand (Figure 1b). It appears that the insertion of the extra Val is responsible for the insolubility but it remains unknown whether it is specifically due to the large hydrophobic side chain of the Val residue or the inserted space.

This insoluble V22-SH3 mutant thus offers an attractive model for detailed CD and NMR characterization. In the present study, we first investigated the conformational properties of V22-SH3 and assessed the consequence of the insertions of other amino acids at the same position by replacing Val22 with Ala, Asp or Arg. Subsequently we conducted an extensive NMR structural and dynamic characterization of V22-SH3 solubilized in salt-free water. The study not only leads to the establishment of the first high-resolution structural and dynamic picture of an insoluble protein, but also reveals the mechanism to rationalize how the mutation/posttranslational modification causes aggregation of disease-related proteins *in vivo*.

## Materials and Methods

### Generation of SH3-1 Mutants

The cDNA encoding V22-SH3 was previously constructed by PCR-based *de novo* gene synthesis [10]. A22-, D22- and K22-SH3 mutants were successfully obtained by QuikChange Site-Directed Mutagenesis Kit (Stratagene, La Jolla, CA, USA) with the DNA oligonucleotides following the previous protocol [11]. All SH3 insertion mutant genes were cloned into the His-tagged expression vector pET32a (Novagen) and their recombinant proteins were over-expressed in *E. coli* strain BL21 cells as previously described [10], [11]. Briefly, the cells were cultured at 37°C to reach an OD_600_ of 0.4, and then IPTG was added to a final concentration of 0.4 mM to induce recombinant protein expression for 12 h at 20°C. V22-, A22-, D22- and K22-SH3 proteins were all found in inclusion body which were not refoldable in buffers. Therefore, they were purified by Ni^2+^ affinity column under denaturing condition in the presence of 8 M urea, followed by further purification by HPLC on a RP-C_18_ column (Vydac).

For NMR isotope labeling, recombinant proteins were prepared by growing the cells in M9 medium with additions of (^15^NH_4_)_2_SO_4_ for ^15^N labeling and (^15^NH_4_)_2_SO_4_ and [^13^C]-glucose for ^15^N and ^13^C labeling, respectively [10], [11]. The identities of the proteins and peptides were verified by MALDI-TOF mass spectrometry.

### Sample Preparation and CD, NMR Experiments

For the soluble wild-type SH3, the CD and NMR samples were prepared by buffer-exchanging the purified protein into 5 mM phosphate buffer at pH 6.2. For V22-, A22-, D22- and K22-SH3-1 mutants, the lyophilized protein powders were dissolved in the deionized water (Millipore, Milli-Q) with addition of an aliquot of 5 mM NaOH to adjust pH and of 10% D_2_O into NMR samples for spin-lock. To minimize the salt introduction which would dramatically reduce the solubility of the four buffer-insoluble SH3 mutants, most CD and NMR experiments were done at pH 4.0. However, for V22-SH3, NMR HSQC experiments were collected at both pH 4.0 and 6.2 for comparison. For ^13^C-HCCH-TOCSY and ^13^C-NOESY experiments, the double-labeled V22-SH3 sample was prepared in D_2_O.

CD experiments were performed on a Jasco J-810 spectropolarimeter equipped with a thermal controller as described previously [10], [11]. The far-UV CD spectra were collected at a peptide concentration of ∼20 µM at 25°C, using 1 mm path length cuvette with a 0.1 nm spectral resolution. The near-UV CD spectra were collected at a protein concentration of ∼200 µM in the absence and in the presence of 8 M urea. Data from five independent scans were added and averaged.

NMR experiments were acquired on an 800 MHz Bruker Avance spectrometer equipped with pulse field gradient units at 298 K as described previously [10], [11], [19]. For the wild-type SH3 domain in the native condition and in the presence of 8 M urea, only a pair of triple-resonance experiments [HNCACB, CBCA(CO)NH, HNCO] were collected for backbone assignment. For V22-SH3, NMR spectra were acquired for both backbone and side chain assignments which included ^15^N-edited HSQC-TOCSY, HSQC-NOESY and ^13^C-HCCH-TOCSY as well as triple-resonance experiments [HNCACB, CBCA(CO)NH, HNCO]. NOE connectivities were identified from ^15^N- and ^13^C-edited NOESY spectra.

NMR data were processed with NMRPipe [20] and subsequently analyzed and fitted by use of NMRView [21]. The published solution structure of the first Nck2 SH3 (2B86) was obtained from PDB and its associated NMR data with accession code of 6854 was downloaded from BioMagResBank [18]. The structure display and analysis were achieved by the graphic software MolMol [22].

### NMR ^15^N Backbone Dynamics

^15^N T1 and T2 relaxation times and {^1^H}-^15^N steady-state NOEs were determined on the 800-MHz spectrometer at 298 K as described previously [10], [23], [24]. ^15^N T1 values were measured by fitting HSQC spectra recorded with relaxation delays of 10, 100, 200, 300, 400, 500, 600 and 700 msec. ^15^N T2 values were determined with relaxation delays of 10, 60, 100, 130, 160, 200, 230, 260 and 300 msec. {^1^H}–^15^N steady-state NOEs were obtained by recording spectra with and without ^1^H presaturation of duration 3 sec plus a relaxation delay of 5 sec at 800 MHz.

The ^15^N relaxation data were analyzed by direct mapping of the reduced spectral density with simplified approximations [25]–[27]. Briefly, J(0), J(ωN), and J(0.87ωH), the spectral densities at the frequencies 0, ωN, and 0.87ωH respectively, were calculated based on Eqs. (1)–(4) [27]–[28]:
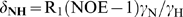
(1)

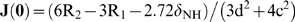
(2)

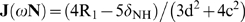
(3)

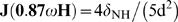
(4)


where 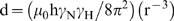 and 
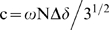
.

## Results

### CD and NMR HSQC Characterization

While the NMR structure of the wild-type first Nck2 SH3 domain was previously determined in 50 mM sodium phosphate buffer (pH 6.5) [18], the same SH3 domain we constructed with a Val insertion was highly insoluble and only found in the inclusion body. Other three insertion mutants with Val22 replaced by Ala, Asp and Arg were also highly insoluble. Furthermore, attempts to refold them by either fast dilution or dialysis in several buffers all failed because recombinant proteins precipitated immediately upon removal of urea, indicating that they were neither refoldable nor insoluble in buffers. As such, the V22-, A22-, D22- and K22-SH3 proteins were purified by Ni^2+^-agrose affinity column under denaturing condition followed by further HPLC purifications on a RP-C_18_ column. The lyophilized V22-, A22-, D22- and K22-SH3 proteins were not soluble in buffer but again could be solubilized in salt-free water.

**Figure 2:**
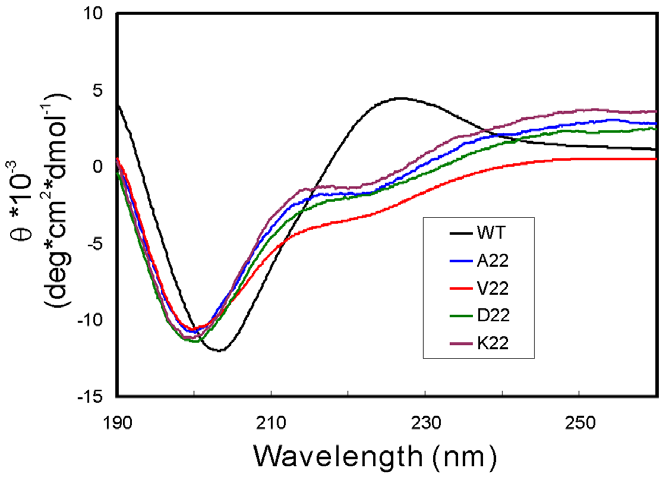
CD characterization. Far-UV CD spectra of the wild-type SH3 and its insertion mutants which were collected at protein concentrations of ∼20 µM at 25°C on a Jasco J-810 spectropolarimeter. The wild-type SH3 was dissolved in 5 mM phosphate buffer (pH 6.2) while the four insertion mutants were solubilized in salt-free water (pH 4.0).

Figure 2 presents far-UV CD spectra of all SH3 domains. The wild-type SH3 domain appears well-folded, with positive CD signals at both ∼190 and ∼228 nm which is characteristic of a β-hairpin/turn dominant protein. By contrast, all four insertion mutants have similar CD spectra with both positive signals lost as well as the maximal negative signal shifted from ∼204 nm to ∼200 nm, indicating that they are largely unstructured. However, the appearance of small negative signals at ∼222 nm indicates that the helical conformation may be weakly-populated [10], [11]. To assess their tertiary packing as previously-described [10], [11], we also acquired the near-UV spectra for all SH3 proteins in the absence and in the presence of 8 M urea (spectra not shown). For the wild-type SH3 domain, a dramatic difference was observed for the near-UV spectra under native and denaturing conditions, indicating that it is well-packed in the native condition. By contrast, no significant difference was found for four insertion mutants under the two conditions, suggesting that their tight packing has been severely disrupted even without the presence of any denaturant [10].

**Figure 3:**
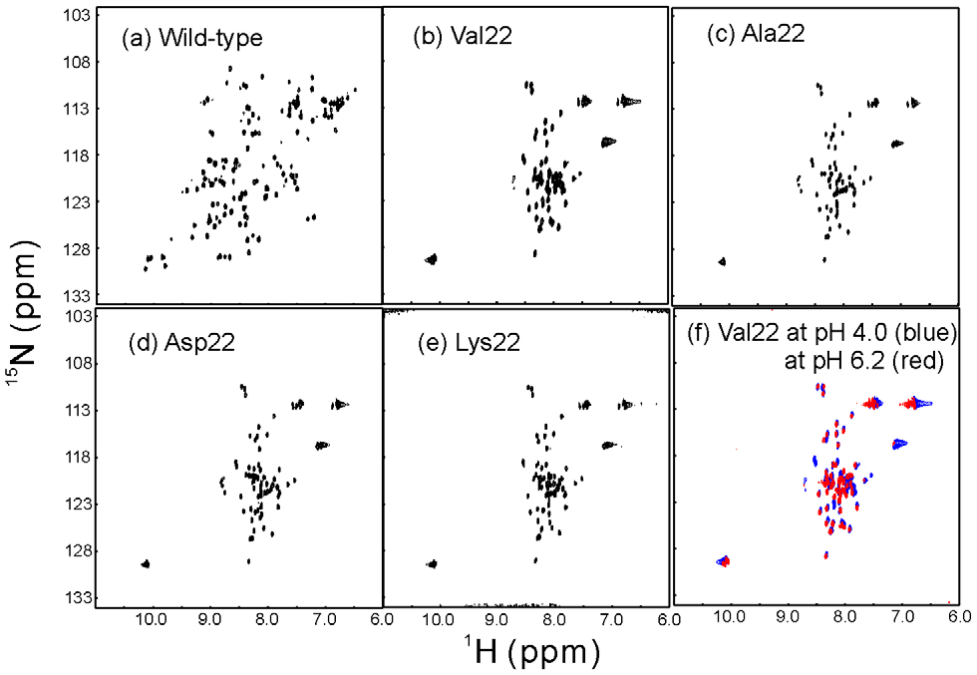
NMR characterization. ^1^H-^15^N NMR HSQC spectra of the wild-type SH3 and its insertion mutants with protein concentrations of ∼100 µM acquired on an 800 MHz NMR spectrometer at 25°C. The wild-type SH3 was dissolved in 5 mM phosphate buffer (pH 6.2) while the insertion mutant were solubilized in salt-free water (pH 4.0). (f). Superimposition of the HSQC spectra of V22-SH3 at pH 4.0 (blue) and at pH 6.2 (red).

In agreement with the CD results, ^1^H-^15^N HSQC spectra again demonstrate that the wild-type SH3 domain is well-folded as evidenced from its large spectral dispersion expected for a well-folded protein (∼3.2 ppm over ^1^H and ∼21 ppm over ^15^N dimensions) (Figure 3a). By contrast, HSQC spectral dispersions are markedly abolished for all four insertion mutants, with only ∼0.85 ppm over ^1^H and ∼18 ppm over ^15^N dimensions (Figure 3b–3e) [29], [30]. We also collected HSQC spectra for V22-SH3 at pH 4.0 and 6.2 at a protein concentration of ∼100 µM, and interestingly their spectral dispersions are essentially very similar (Figure 3f). However, at pH 6.2, the severe NMR line broadening took place at higher protein concentrations (spectra not shown). Based on these results, it is feasible to conclude that the insolubility of V22-SH3 is not directly owing to the introduction of the large Val hydrophobic side-chain. Instead, it may be the insertion of one residue space at the tip of the diverging turn which results in insolubility.

We have also performed NMR hydrogen/deuterium exchange experiments on V22-SH3 but failed to identify any slowly-exchanged amide proton even at pH 4.0 (data not shown). This result together with near-UV and NMR HSQC spectra, indicate the tight side chain packing is severely disrupted in V22-SH3 and consequently the whole protein sequence is considerably accessible to the bulk solvent.

### Chemical Shift Deviations

**Figure 4:**
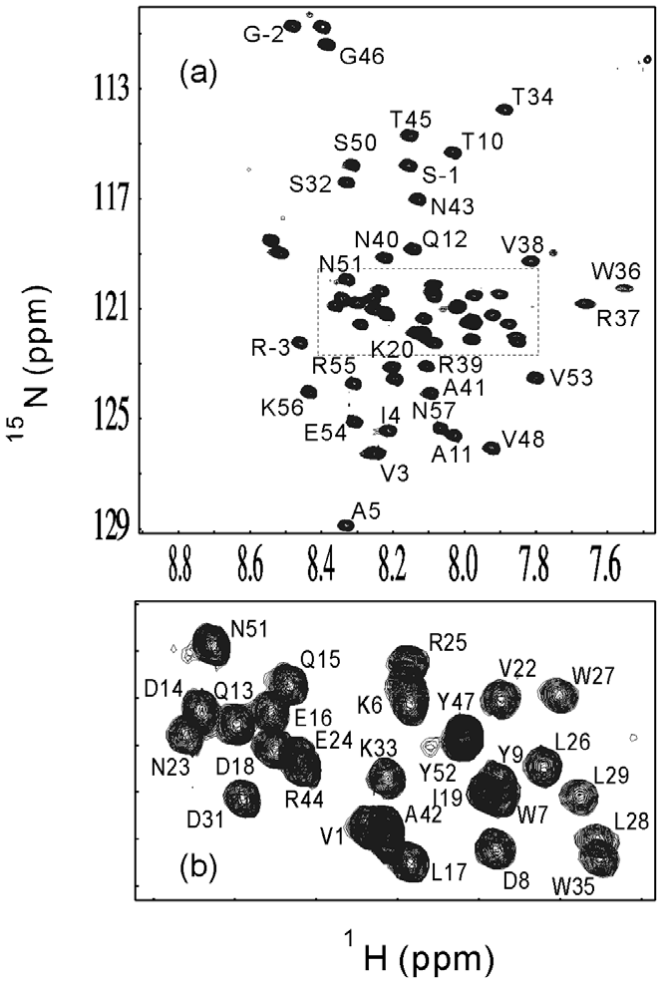
NMR sequential assignment. ^1^H-^15^N NMR HSQC spectrum of V22-SH3 with a protein concentration of ∼800 µM dissolved in salt-free water (pH 4.0) acquired on an 800 MHz NMR spectrometer at 25°C. The assigned residues are labelled in the full (a) and expanded spectra (b).

**Figure 5:**
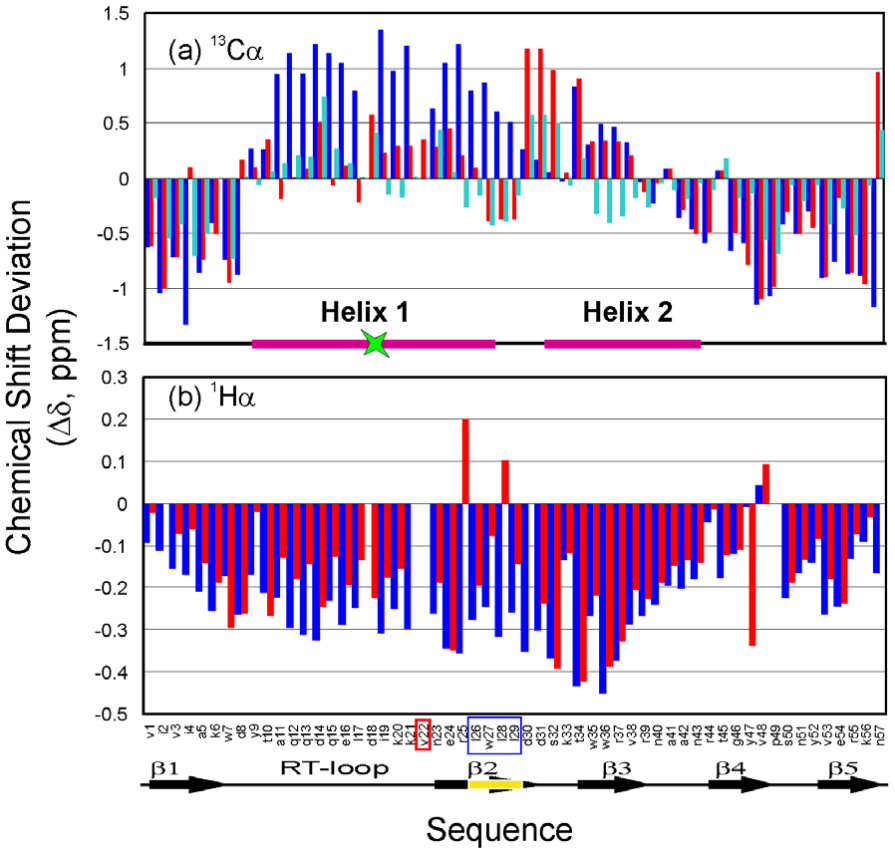
NMR chemical shift deviations. (a) Bar plot of Cα chemical-shift deviations from their random-coil values for the V22-SH3 domain solubilized in salt-free water at pH 4.0 (red); wild-type at pH 2.0 (blue) and wild-type in the presence of 8 M urea (cyan). (b) Bar plot of Hα chemical-shift deviations for the V22-SH3 domain solubilised in salt-free water at pH 4.0 (red) and wild-type at pH 2.0 (blue). Red box indicates the Val22 insertion while blue box indicates previously mutated region. Secondary-structure fragments are also indicated for both the native SH3 fold and non-native helical conformations previously observed [11].

To gain detailed structural and dynamic properties, we further double-labeled V22-SH3 and subsequently conducted an extensive heteronuclear NMR study on it. Although its NMR spectral dispersions were significantly lost and resonance peaks were largely-degenerated, we have succeeded in NMR assignments of the insoluble V22-SH3 domain solubilized in salt-free water (Figure 4) and calculated Cα and Hα chemical shift deviations from their random coil values. It has been well-established that these deviations are very sensitive indicators of protein secondary structures, thus representing a powerful probe to detect residual secondary structures in unfolded or partially-folded proteins [29]–[35]. Figure 5 presents the Cα and Hα chemical shift deviations of V22-SH3. Overall, the deviations are relatively small as compared to those for a typically-folded protein, thus indicating that V22-SH3 is largely unfolded. More specifically, as seen in Figure 5, based on the Cα and Hα chemical shift deviations, it appears that in V22-SH3, the N- (residues 1–6) and C- (residue 47–57) termini are largely unstructured, without any significant secondary structure populated. On the other hand, previously we have demonstrated that the non-native helical conformations were highly populated over two regions, residues 7–21 and 27–45 in the wild-type SH3 domain at pH 2.0 and 4Ala mutant at pH 6.5 [11]. Very interestingly, here as judged from the chemical shift deviations, it seems that in V22-SH3, the non-native helical conformation is highly populated over the secondary region but no significant secondary structure preference is populated over the first region.

### NOE Analysis

To gain insights into packing properties, we have acquired both ^15^N- and ^13^C-edited NOESY spectra for the V22-SH3 domain solubilized in salt-free water at a protein concentration of ∼800 µM. As shown in Supplementary Figure S1, many NOE connectivities are observed in two NOESY spectra. For example, long-range NOEs can be identified between protons of the well-resolved Trp side-chain and other residues (Supplementary Figure S1b). More importantly, these NOEs can be assigned as exemplified in Supplementary Figure S1c. The assignment results reveal that many residues still have medium-range NOEs and strikingly 23 residues (∼40% of the molecule) even own long-range NOEs. Usually it is very difficult to observe long-range in unfolded or partly folded proteins, due to their intrinsic flexibility and poor resonance dispersion. However, observation of long-range NOEs between protons provides definitive evidence that they are in close proximity in at least some structures in the conformational ensemble [30].

**Figure 6:**
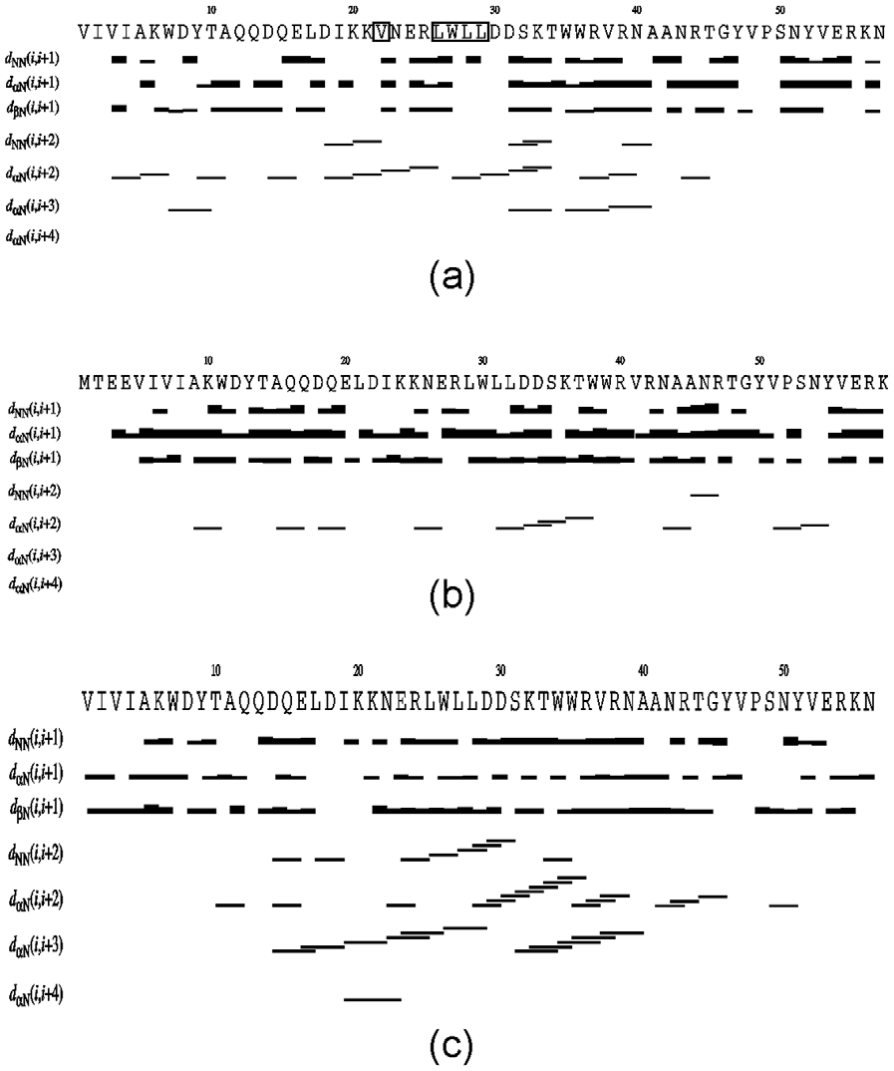
Characteristic NOEs defining secondary-structures. NOE connectivities identified for (a) V22-SH3 solubilized in salt-free water at pH 4.0; (b) wild-type in 50 mM sodium phosphate buffer (pH 6.5) previously published [18]; and (c) wild-type in 5 mM phosphate buffer (pH 2.0) [11]. Plots were generated by CYANA 2.1.

A detailed comparison of the NOEs identified in V22-SH3 here with those associated with the published NMR structure of the wild-type SH3 domain [18] leads to classification of NOEs into two categories: native-like and non-native (Supplementary Table S1 and S2). As shown in Supplementary Table S2 and Figure 6a, non-native NOEs are all sequential and medium-range manifested over almost all the sequence. As seen in Figure 6b, for the wild-type SH3 domain in the native condition, there are only limited amount of αH(i)-NH(i+2) NOEs over the loop and turn regions but no αH(i)-NH(i+3) and αH(i)-NH(i+4) NOEs at all, compatible with its β-barrel native structure. By contrast, in the wild-type SH3 at pH 2.0 (Figure 6c), there are many non-native αH(i)-NH(i+3) NOEs manifested over two regions which were previously characterized to adopt highly-populated helical conformations. In V22-SH3, although the number largely reduces, there are still many non-native αH(i)-NH(i+2) and αH(i)-NH(i+3) NOEs over the sequence, in particular over residues 28–42, corresponding to the second helical region in the wild-type SH3 at pH 2.0 [11]. These NOEs are totally incompatible with the well-form and rigid β-barrel structure of the SH3 domain determined by NMR in the native condition [18]. As such, the manifestation of these non-native medium-range NOEs is in a nice agreement with the chemical shift deviations suggesting that in V22-SH3, the non-native helical conformation is also highly populated over the second region, but not the first region. In the meanwhile, many native medium-range NOEs over the β-turn/loop regions are also preserved, indicating that these turns/loops are still populated in V22-SH3.

**Figure 7:**
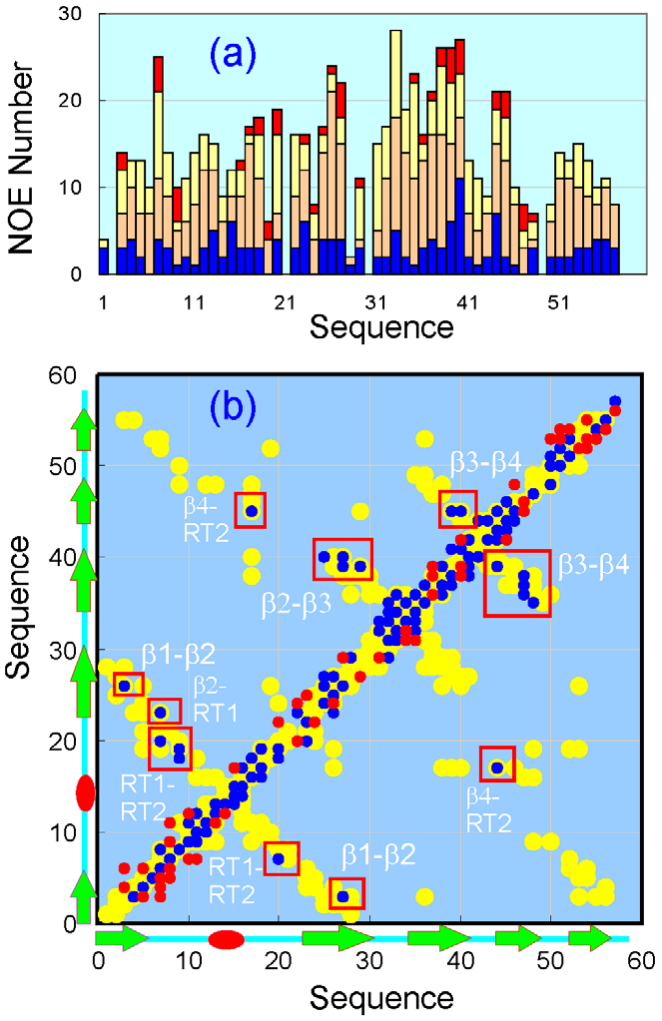
NOE plots. (a). NOE numbers identified from a ^15^N-HSQC-NOESY spectrum of V22-SH3. Blue bars: intra-residual NOEs; brown bars: sequential NOEs; yellow bars: medium-range NOEs and red bars: long-range NOEs. (b). NOE contact map: yellow spots: NOEs of the wild-type SH3 domain associated with the published NMR structure determined at pH 6.5 [18]. Blue spots: native-like NOEs of V22-SH3. Red spots: non-native NOEs of V22-SH3. The persistent long-range NOEs were highlighted by red boxes.

Previously, no long-range NOEs could be identified in the wild-type SH3 at pH 2.0 and 4Ala mutant at pH 6.5, both of which own highly populated helical conformation [11]. However, in V22-SH3, there still exist many native-like long-range NOEs as summarized in Figure 7 and Supplementary Table S1. More specifically, except for the fifth β-strand having no long-range NOE, β-strands 1–4 as well as the two RT-loop strands are found to have native long-range NOEs (Figure 7b). For example, 2 native-like long-range NOEs still persist between the first and second β-strands, 6 between the second and third strands, 7 between the third and fourth strands, and 7 between the two RT-loop strands. Therefore, these persistent native-like long-range NOEs imply that despite severely-disrupted tight packing and populated non-native secondary structures, at least some structures in the conformational ensemble of the partially-folded V22-SH3 still have a rudiment tertiary topology similar to its native SH3 fold [18].

### NMR ^15^N Relaxation Data and Reduced Spectral Density Mapping

**Figure 8:**
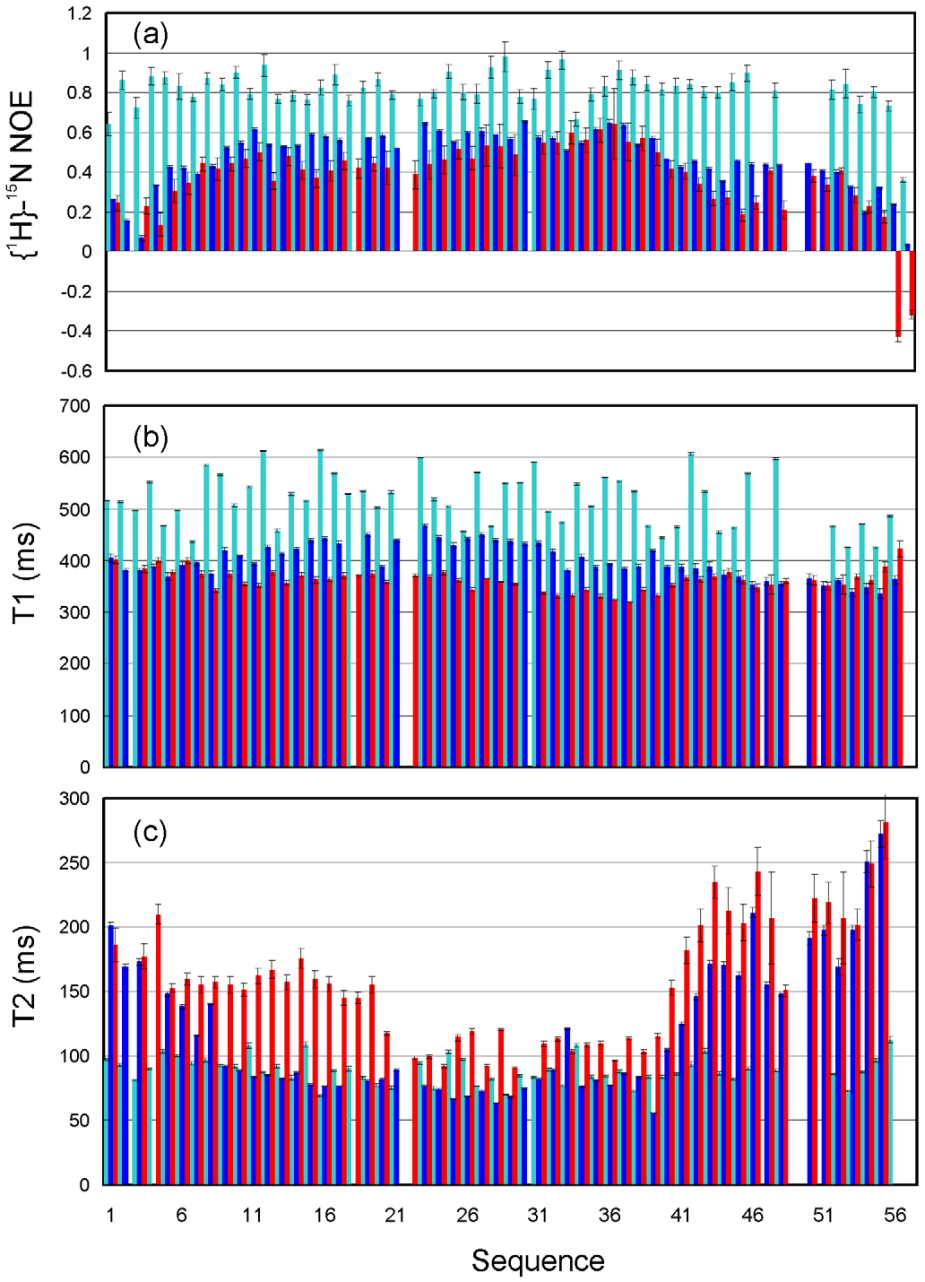
^15^N NMR backbone relaxation data. The ^15^N NMR backbone relaxation data of V22-SH3 solubilized in salt-free water at pH 4.0 (red); wild-type at pH 6.5 (cyan); and wild-type at pH 2.0 (blue) as measured at 800 MHz. (a). {^1^H}-^15^N steady-state NOE intensities. (b). ^15^N T1 (longitudinal) relaxation times. (c) ^15^N T2 (transverse) relaxation times.

We also collected ^15^N NMR backbone relaxation data for V22-SH3 which were very enlightening to pinpoint the dynamics of the local environment of a protein on the pico- to nano-second timescale. In particular, {^1^H}-^15^N steady-state NOE (hNOE) offers a measure to the backbone flexibility [23]–[30]. As seen in Figure 8a, if compared with the wild-type SH3 domain at pH 6.5, V22-SH3 has significantly-reduced hNOE values over the whole sequence, in particular over the N- and C-termini which are characterized above to be highly unstructured. Nevertheless, except for the C-terminal two residues, all V22-SH3 residues still have positive hNOE values, with many >0.4. In particular, hNOE values>0.6 are found for two residues, Trp35 and Trp36, which are located at the central positions of the region that is above characterized to own a highly populated helical conformation.

**Figure 9:**
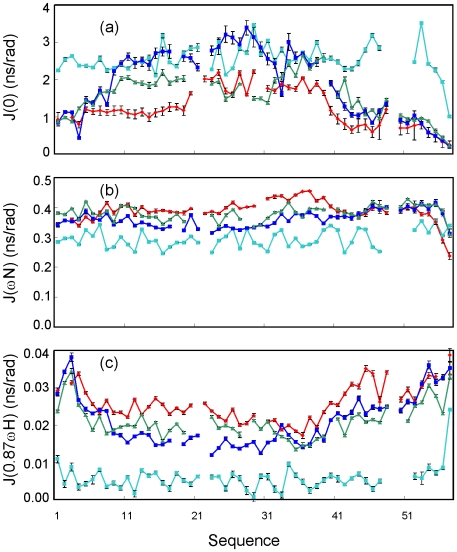
Reduced spectral density functions. Spectral densities of V22-SH3 solubilized in salt-free water at pH 4.0 (red); wild-type at pH 6.5 (cyan); wild-type at pH 2.0 (blue) and 4Ala mutant at pH 6.5 (green), calculated from the ^15^N backbone relaxation data measured at 800 MHz. (a) J(0), (b) J(ωN), and (c) J(0.87ωH).

Furthermore, we also calculated reduced spectral densities (Figure 9) at three frequencies, ω0, ωN and 0.87ωH, from the ^15^N backbone relaxation data at 800 MHz, which reflect relaxation contributions from the motions on different timescales [23]–[30], [36]. As seen in the equations 1–4 in the Materials and Methods, rapid internal motions on the ps-ns timescale tend to reduce the value of J(0), while slow motions on the µm-ms time scale lead to large values of J(0). On the other hand, the high-frequency spectral density J(0.87ωH) is only sensitive to fast internal motions which will result in relatively large values of J(0.87ωH).

As seen in Figure 9, while J(ωN) is much less informative, J(0) and J(0.87ωH) offer critical insights into the motion variations in V22-SH3. As shown in Figure 9c, if compared to the wild-type SH3 domain at pH 6.5, V22-SH3, wild-type SH3 at pH 2.0 and 4Ala mutant all have significantly increased J(0.87ωH) over the whole sequence, indicating that a dramatic increase in the fast motions on the ps-ns timescale for these largely-unfolded SH3 forms. Interestingly, out of three largely-unfolded SH3 forms, V22-SH3 uniformly has the highest J(0.87ωH) values, suggesting that V22-SH3 has the largest increase of the fast motions. On the other hand, in V22-SH3 large J(0.87ωH) and small J(0) over the N- and C-termini indicate that the termini are more flexible than other regions. Interestingly, in V22-SH3, the region with the smallest J(0.87ωH) is over residues Lys33-Thr34-Trp35-Trp36 (Figure 9c), which are located in the central part of the region with a highly-populated helical conformation. It is particularly interesting to note that although residues Lys33-Thr34-Trp35-Trp36 with the smallest J(0.87ωH) do have relatively large J(0) values, the region with the largest J(0) values are over Glu24-Arg25-Leu26-Trp27-Leu-28-Leu29 (Figure 9a). This observation implies that slow motions on the µm-ms time scale or/and dynamic aggregation also contribute to the J(0) over this region. Strikingly, this region was previously revealed to play a critical role in coordinating the transformation from the non-native helical conformation to native all-β SH3 fold during the folding of the first hNck2 SH3 domain [11].

### Salt Effect on the Solubility of V22-SH3

**Figure 10:**
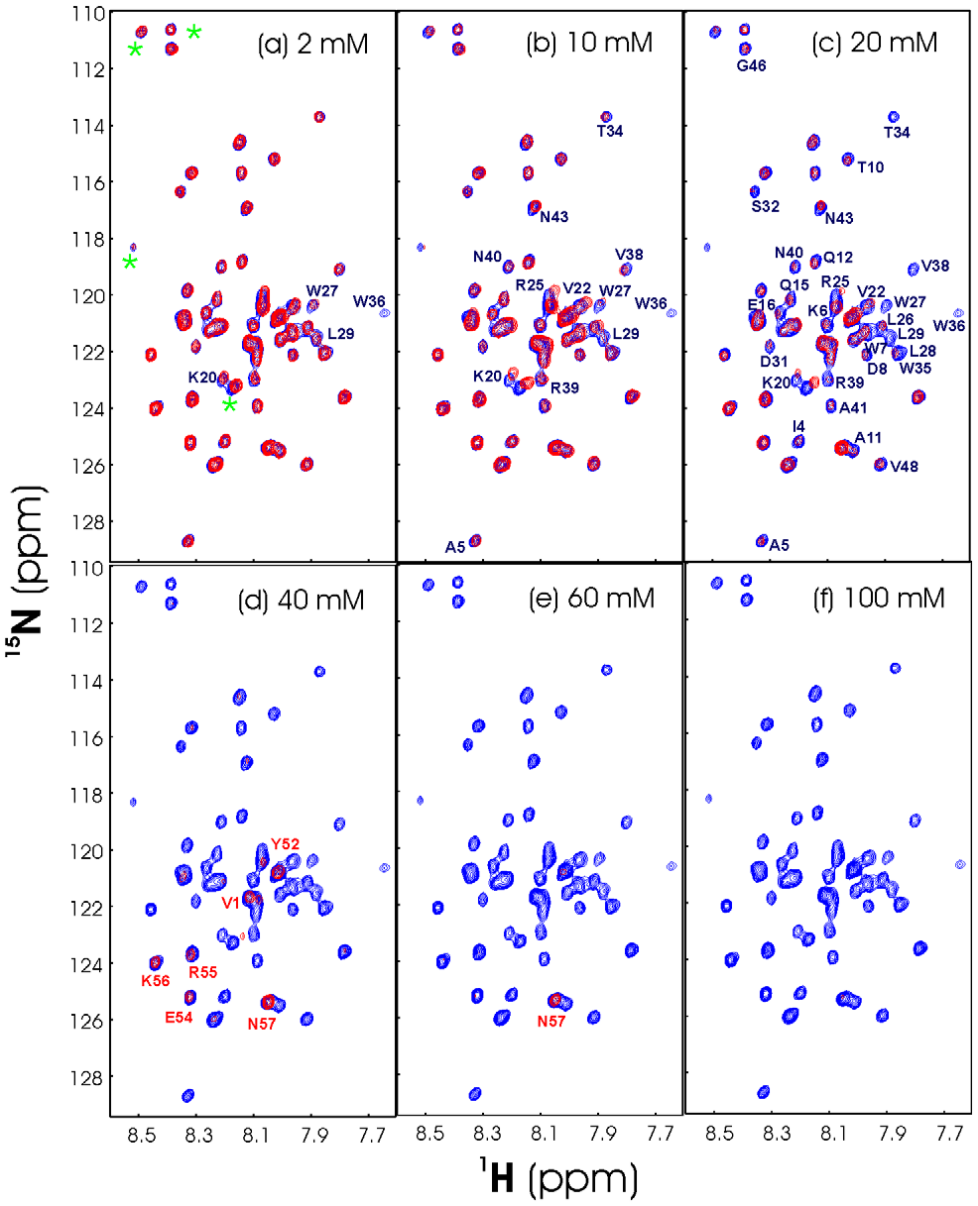
Salt titrations as monitored by HSQC. Superimposition of the ^1^H-^15^N NMR HSQC spectra of V22-SH3 at a protein concentration of ∼50 µM, solubilized in salt-free water (pH 4.0) (blue), and with additional introduction of NaCl (red) to 2 mM (a); 10 mM (b); 20 mM (c); 40 mM (d); 60 mM (e); and 100 mM (f). The HSQC spectra were acquired on an 800 MHz NMR spectrometer at 25°C. The blue font is used for labelling the residue with its HSQC peak intensity significantly reduced or disappeared, while the red is for the residue with its HSQC peak still observed. *: HSQC peaks resulting from the residues located on the His-tag.

To address how the salt concentration affects the solubility of V22-SH3, we have conducted extensive titrations of NaCl into various V22-SH3 samples solubilized in salt-free water. If the V22-SH3 concentration is high (>300 µM), addition of NaCl even to 5 mM would result in visible aggregation rapidly. As such, in order to monitor the aggregation process by NMR HSQC experiments, we lowered the V22-SH3 concentration down to ∼50 µM and subsequently collected a series of HSQC spectra by gradually increasing the NaCl concentrations. As shown in Figure 10, overall, addition of NaCl caused no significant shift of the HSQC peaks of V22-SH3, convincingly demonstrating that no fundamental difference exists for its conformations in the absence and presence of salt. However, although no visible aggregate was observed during the experiments, addition of salt even to 2 mM induces the NMR line broadening which leads to the disappearance of HSQC peaks (Figure 10a). This implies that addition of salt even to a very low concentration induces dynamic aggregation or conformational exchanges on the µs-ms time scale. As seen in Figure 10d, at a NaCl concentration of 40 mM, most HSQC peaks disappear except for those of several C-terminal residues. When the NaCl concentration reaches 100 mM, all peaks become too broad to be detected (Figure 10f). Moreover, after more than 5 hours, the visible aggregates even formed in the V22-SH3 sample in the presence of only 5 mM NaCl.

## Discussion

Previous inability to solubilize insoluble protein without adding denaturants and detergents retarded our complete understanding of the mechanisms underlying protein folding and disease-relevant aggregation because many folding intermediates and disease-causing mutants are highly insoluble. Recently we have discovered that protein insolubility could be overcome by suppressing attractive hydrophobic interactions with intrinsically repulsive electrostatic interactions which are expected to have the largest strength in salt-free water [4]–[11].

In the present study, we have used this approach again to successfully solubilize the insoluble V22-SH3 and three other insertion mutants, and show that all four insertion mutants are largely unfolded without stable secondary structures and tight tertiary packing by quick CD and NMR characterization. Furthermore, we demonstrate that it is most likely that the one residue space inserted, rather than the specific properties of the inserted residue, causes the significant unfolding and insolubility, because all four insertion mutants (V22-, A22-, D22- and R22-SH3) are similarly unfolded and insoluble in buffers. Interestingly, the hNck2 sequence AAC04831 was originally cloned from a tumor tissue and as such it will of significant interest to explore whether this insertion plays any role in tumorigenesis in the future.

Furthermore, our thorough NMR characterization leads to the establishment of a high-resolution structural and dynamic picture for the insoluble V22-SH3 domain solubilized in salt-free water. In V22-SH3, except for a small region Ser32-Asp40 with highly-populated helical conformation, the rest of the protein appears to have no significant secondary structure preference. Most strikingly, on the other hand, a small set of native-like long-range NOEs still preserves in V22-SH3 which imply that at least some structures in the conformational ensemble still have a rudiment tertiary topology similar to its native SH3 fold [18], [30]. By contrast, previously in the wild-type SH3 at pH 2.0 and 4Ala mutant at pH 6.5, the non-native helical conformations were found to be highly-populated over almost the whole sequence except for the N- and C-termini but in the meanwhile no long-range NOEs could be observed.

The nonhierarchical mechanism proposes that the folding of β-proteins may follow two major transitions. The first is involved in the collapse of the random-coil-like polypeptide chain into a nonnative helical intermediate mainly specified by local interactions, whereas the second is associated with the transformation into the native β-structure, with the helical conformation disrupted by long-range interactions [37]–[43]. Previously we have proposed that in wild-type SH3 domain at pH 2.0 and 4Ala mutant at pH 6.5 [11], the folding was trapped at the first stage in which no significant tertiary packing was in place but the non-native helical conformations are highly-populated over the whole sequence except for the N- and C-termini. Furthermore, we also speculated that the second folding transition of the SH3 domain might be mainly coordinated by forming the tertiary packing around the 4 residues Leu26-Trp27-Leu28-Leu29 [11]. Our present results enforce this speculation because a rudimentary native-like tertiary packing is populated in V22-SH3 which owns those 4 residues. However, due to the insertion at the tip of the divergent turn, V22-SH3 seems trapped in the middle of the second transition, in which the non-native helical conformations are largely disrupted and a native-like tertiary packing core is populated to some extent. In V22-SH3, the final secondary structures as well as specific and tight tertiary packing have not yet been formed probably due to the failure of the correct formation of the divergent turn. Interestingly, as seen in the sequence alignment of a large array of SH3 domains, four residues involved in forming the divergent turn are found to be mostly polar residues (Supplementary Figure S2). Therefore, on the one hand, our result strongly underscores the critical role of the diverging turn in folding of the SH3 domain, as previously proposed from extensive experimental and simulation studies [44]–[54]. Moreover, it is also implied that the overall fold of a protein may be mainly maintained by a subset of hydrophobic residues, as previously shown for molten globules, but the final formation of the native structure needs precise and specific packing also involved in hydrophilic residues [55]–[68].

Our study also provides a mechanism to explain why one residue insertion at the divergent turn is sufficient to cause the SH3 domain totally insoluble. It appears that in the 4Ala mutant, the absence of four residues Leu26-Trp27-Leu28-Leu29 traps the folding at the first stage, with the helical conformations highly-populated over the SH3 domain. However, the formation of the helical conformations will allow the proper burial of most hydrophobic side chains, thus preventing aggregation of the 4Ala mutant in buffers even at pH 6.5. By contrast, in V22-SH3, the helical conformation over the Trp7-Lys21 is largely disrupted by the presence of four residues Leu26-Trp27-Leu28-Leu29, but the specifically-packed native structure has not been formed yet because of one residue insertion at the divergent turn. As such, in V22-SH3, many hydrophobic side chains are exposed and also largely accessible to bulk solvent, as supported by the observation that no slowly-exchanged amide proton could be identified and out of three largely-unfolded SH3 forms, V22-SH3 has the least-restricted backbone motions. Consequently, if V22-SH3 is in buffers, the salt ions will screen out the repulsive electrostatic interactions and thus allow the hydrophobic interactions to dominate. This will lead to an immediate aggregation of the V22-SH3 domain in buffers.

Here we propose that the same mechanism may also be underlying the *in vivo* aggregation of the disease-related proteins with the genetic mutation or posttranslational modification. More specifically, the mutation or modification of these proteins may trap them in highly unstructured or partially-folded states with a large portion of hydrophobic side chains exposed/accessible to bulk solvent. In the physiological condition, the salt concentration is ∼150 mM which is sufficient to largely screen out the repulsive electrostatic interactions, thus resulting in a severe aggregation *in vivo*. Finally, our present success again highlights the promising potential to use salt-free water to solubilize various previously thought insoluble proteins for high-resolution biophysical investigations to better understand mechanisms for protein folding and aggregation responsible for a large array of human diseases [1]–[4], [68]–[70].

## Supporting Information

Figure S1 NOE identification and assignment (a) 13C-edited NOESY spectrum of the V22-SH3 domain with a protein concentration of ∼1 mM collected in salt-free D2O (pD 4.0) at 25°C. (b) 15N-edited HSQC-NOESY spectrum of the V22-SH3 domain collected in salt-free water (pH 4.0) at 25°C. The NOE connectivities between the ring NH of Trp residues and other protons were indicated by arrows. Both spectra were collected on an 800 MHz Bruker Avance NMR spectrometer. (c) Strips of HSQC-TOCSY and HSQC-NOESY spectra to exemplify the assignment of the long-range NOEs.
(4.23 MB TIF)

Figure S2 Alignment of the representative SH3 sequences Green box is used for indicating 4 residues forming the diverging turn of SH3 domains, linking the RT-loop and the second beta-strand (Figure 1b), while red box is used to indicate the four residues previously revealed to play a critical role in coordinating the transformation from the non-native helical conformation to native all-beta fold during the folding of the first hNck2 SH3 domain [11].
(1.50 MB PDF)

Table S1 Native-like long-range NOEs persistent in V22-SH3
(0.03 MB DOC)

Table S2 Non-native NOEs identified in V22-SH3
(0.03 MB DOC)
