# Supplementary figures and images for: Insights into Protein Aggregation by NMR Characterization of Insoluble SH3 Mutants Solubilized in Salt-Free Water
Source: PLoS One. 2009 Nov 23;4(11):e7805. doi: 10.1371/journal.pone.0007805 (PMC2776303; doi:10.1371/journal.pone.0007805)

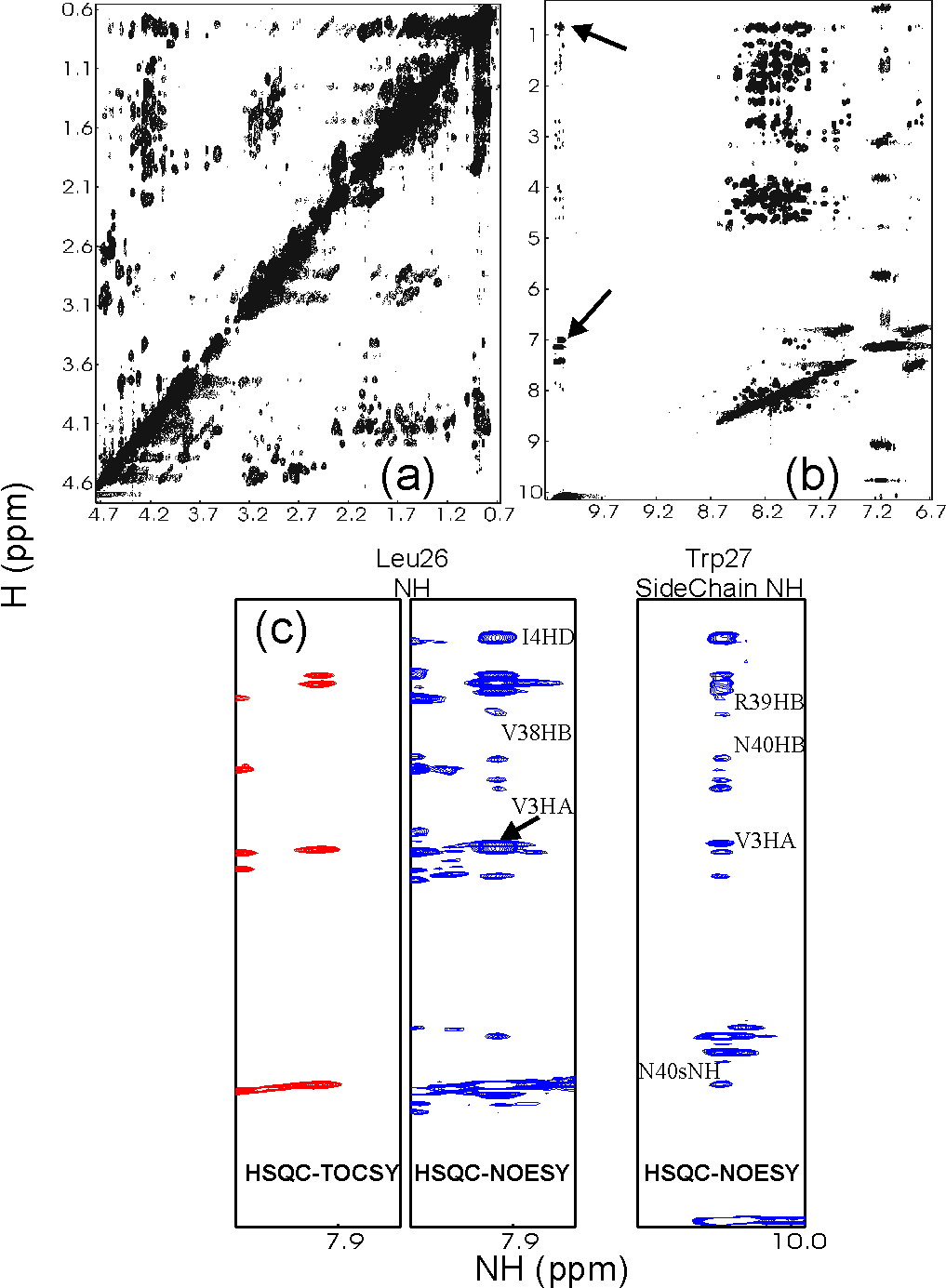

Supplement: Figure S1 — NOE identification and assignment (a) 13C-edited NOESY spectrum of the V22-SH3 domain with a protein concentration of ∼1 mM collected in salt-free D2O (pD 4.0) at 25°C. (b) 15N-edited HSQC-NOESY spectrum of the V22-SH3 domain collected in salt-free water (pH 4.0) at 25°C. The NOE connectivities between the ring NH of Trp residues and other protons were indicated by arrows. Both spectra were collected on an 800 MHz Bruker Avance NMR spectrometer. (c) Strips of HSQC-TOCSY and HSQC-NOESY spectra to exemplify the assignment of the long-range NOEs. (4.23 MB TIF) [file pone.0007805.s001.tif]
