# Supplementary material for: Insights into Protein Aggregation by NMR Characterization of Insoluble SH3 Mutants Solubilized in Salt-Free Water
Source: PLoS One. 2009 Nov 23;4(11):e7805. doi: 10.1371/journal.pone.0007805 (PMC2776303; doi:10.1371/journal.pone.0007805)
Supplement: Figure S2 — Alignment of the representative SH3 sequences Green box is used for indicating 4 residues forming the diverging turn of SH3 domains, linking the RT-loop and the second beta-strand (Figure 1b), while red box is used to indicate the four residues previously revealed to play a critical role in coordinating the transformation from the non-native helical conformation to native all-beta fold during the folding of the first hNck2 SH3 domain [11]. (1.50 MB PDF) [file pone.0007805.s002.pdf]

Simple
Modular
Architecture
Research
Tool
SMART MODE:

NORMALGENOMIC

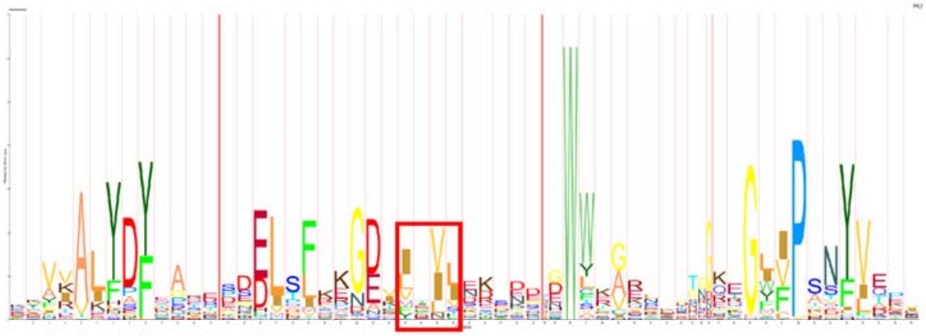

Explanation of colour codes used by CHROMA is available here.

```
O61080/1-59       GPEQARALYD FAA----ENP DELTFNEGAV VTVINKS--- --NPDWWEGE LN------GQ RGVFP---AS YVELIP
SEM5_CAEEL/1-59   ETKFVQALFD FNP----QES GELAFKRGDV ITLINKD--- --DPNWWEGQ LN------NR RGIFP---SN YVCPYN
AAC78611/1-59     AVCQVIGMYD YTA----QND DELAFNKGQI INVLNKE--- --DPDWWKGE VN------GQ VGLFP---SN YVKLTT
O08757/1-59       SQLVVRAKFN FQQ----TNE DELSFSKGDV IHVTRVE--- --EGGWWEGT HN------GR TGWFP---SN YVREIK
O00851/1-56       KLPQVKALYP YTA----AND EELSFKVGDI ITILEK---- --DEGWWKGE LN------GQ EGWIP---NN YVKEI-
O08526/1-60       YFGTAKARYD FCA----RDR SELSLKEGDI IKILNKK--- -GQQGWWRGE IY------GR IGWFP---SN YVEEDY
DRK_DROME/1-59    EEMLVQALYD FVP----QES GELDFRRGDV ITVTDRS--- --DENWWNGE IG------NR KGIFP---AT YVTPYH
MYSD_DICDI/1-59   VKPSAKALYD FDA----ESS MELSFKEGDI LTVLDQS--- --SGDWWDAE LK------GR RGKVP---SN YLQLIK
GRB2_XENLA/1-59   QPTYVQALFD FDP----QED GELGFRRGDF IQVVDNS--- --DPNWWKGT CL------SQ TGMFP---RN YVTPVN
P70297/1-59       EGRKVRAVYD FEA----AED NELTFKAGEI ITVLDDS--- --DPNWWKGE TH------QG VGLFP---SN FVTADL
O01498/1-59       PEKNVRALYD FEA----AES NELSFVAGDI ITITDES--- --NPHWWTGR IG------TQ QGLFP---SS FVTNQL
GRAP_HUMAN/1-59   GACFAQAQFD FSA----QDP SQLSFRRGDI IEVLERP--- --DPHWWRGR SC------GR VGFFP---RS YVQPVH
HCK_HUMAN/1-60    EDIIVVALYD YEA----IHH EDLSFQKGDQ MVVLEE---- --SGEWWKAR SLA----TRK EGYIP---SN YVARVD
63896/1-61        GVTLFIALYD YEA----RTE DDLSFQKGEK FHIINNT--- --EGDWWEAR SLS----SGA TGYIP---SN YVAPVD
YHA2_YEAST/1-59   VVRRVRALYD LTT----NEP DELSFRKGDV ITVLEQV--- --YRDWWKGA LR------GN MGIFP---LN YVTPIV
O74749/1-59       TVSRVRALYD FAA----TEQ GELSFKKGDI ILVLESV--- --YKDWWKGS CK------NA VGIFP---VN YVQRVV
YG3D_YEAST/1-59   TEEYVEALYD FEA----QQD GDLSLKTGDK IQVLEKI--- --SPDWYRGK SN------NK IGIFP---AN YVKPAF
SRK1_SPOLA/1-62   KYPIYVGKYD YDS----RTD DDLSFKKGDL MYIISTD--- --EGDWWFAR SKD---TAGK EGYIP---SN YVAEYK
Q61745/1-68       HGQYFVALFD YQA----RTA EDLSFRAGDK LQVLDTS--- --HEGWWLAR HLE(6)GQQL QGYIP---SN YVAEDR
O76049/1-59       YIEKVVAIYD YTK----DKD DELSFMEGAI IYVIKKN--- --DDGWYEGV CN------RV TGLFP---GN YVESIM
Q62422/1-59       QVKVFRALYT FEP----RTP DELYFEEGDI IYITDMS--- --DTSWWKGT CK------GR TGLIP---SN YVAEQA
Q99962/1-59       DQPCCRALYD FEP----ENE GELGFKEGDI ITLTNQI--- --DENWYEGM LH------GH SGFFP---IN YVEILV
O13064/1-60       QGNIVIALYP YQG----IHE DDLSFKKGEK LKVLEE---- --HGEWWKAK SLS----TKK EGFIP---SN YVARVN
MYSB_ACACA/1-57   AKPQVKALYD YDA----QTG DELTFKEGDT IIVHQKD--- --PAGWWEGE LN------GK RGWVP---AN YVQDI-
Q24218/1-62       VLDIVVALYS FTS----NND QELSFEKGDR LEIVDRPA-- -SDPDWYKAR NN-----QGQ VGLVP---RN YLQELN
O61618/1-59       ILDKVIALYP YKA----QND DELSFDKDDI ISVLGRD--- --EPEWWRGE LN------GL SGLFP---SN YVGPFV
AAD08655/1-59     DGLRAKALYD YQA----TAE DELTFDPNEI ITHVETI--- --DDGWWKGV CR------GK VGLFP---AN YVEMLY
Q63767/1-62       KNVLAKALYD NVA----ESP DELSFRKGDI MTVLERDT-- QGLDGWWLCS LH------GR QGIVP---GN RLKILV
Q24218_1/1-59     AIGTAVVKYN YQA----QQP DELSLTKGTR ILILEKS--- --NDGWWRGQ SG------NS VGWFP---SN YTTEDC
```

```
O62527/1-59       QPLVVQAEYS FMG----SNN DELCFQKGDV ITVTQRE--- --DGGWWEGT LN------DK TGWFP---SN YVNECK
PDB|1GBR|1GBR/1   GSMEAIAKYD FKA----TAD DELSFKRGDI LKVLNEE--- -CDQNWYKAE LN------GK DGFIP---KN YIEMKP
R167_YEAST/1-61   GVETVTALYD YQA----QAA GDLSFPAGAV IEIVQRTP-- -DVNEWWTGR YN------GQ QGVFP---GN YVQLNK
BAA34397/1-59     TGIHAIALYD YQA----ADD DEISFDPDDV ITHIEKI--- --DDGWWRGL CK------NR YGLFP---AN YVQVVG
LCK_CHICK/1-60    QDKLVVALYD YEP----THD GDLGLKQGEK LRVLEE---- --SGEWWRAQ SLT----TGQ EGLIP---HN FVAMVN
AAC78611_1/1-58   SGEEFIAMYT YES----SEQ GDLTFQQGDV ILVTKK---- --DGDWWTGT VG------DK AGVFP---SN YVRLKD
O77050/1-61       EVLTYIALYD YDA----RSE SDLSFKKGEK LEILNNT--- --DGDWWLAK SMV----TGR DGYIP---SN YIAPVK
NCK_HUMAN/1-62    VLHVVQALYP FSS----SND EELNFEKGDV MDVIEKPE-- -NDPEWWKCR KI-----NGM VGLVP---KN YVTVMQ
Q99469/1-59       QMNTYVALYK FVP----QEN EDLEMRPGDI ITLLEDS--- --NEDWWKGK IQ------DR IGFFP---AN FVQRLQ
Q94879/1-61       NVKIFVALYD YDA----RTD EDLSFRKGEH LEILNDT--- --QGDWWLAR SKK----TRS EGYIP---SN YVAKLK
Q23507/1-60       SKTKAVVKFT YEP----RLE DELGLTKGDF VYVVEKS--- --TDGWWKGE AP-----NGG VGWFP---SN YVEEVE
O88903/1-59       KQRQCKVLFD YSP----QNE DELELIVGDV IDVIEEV--- --EEGWWSGT LN------NK LGLFP---SN FVKELE
O42287_1/1-58     EGLQAQALYP WRA----KKD NHLNFNKNDV ITVLEQ---- --QDMWWFGE VQ------GQ KGWFP---KS YVKLIS
Q23507_1/1-62     SFEVVVALYS FDA----SSS EELSFKKGER LEIVDHPE-- -HDPDWWMAR NA-----SGT TGLVP---RN YIEVVV
O42287_2/1-61     KVVYYRALYP FDA----RSH DEITIEPGDI IMVDESQT-- -GEPGWLGGE LK------GK TGWFP---AN YAERMP
AAD00899_1/1-58   VGEEYIALYP YSS----VEP GDLTFTEGEE ILVTQK---- --DGEWWTGS IG------DR SGIFP---SN YVKPKD
AAD03595/1-60     FKCSVRALFD YKA----QRE DELTFTKNTI IQNVEKQ--- --EGGWWRGD CG-----GKK QMWFP---AN YVEEIF
O08635/1-59       GAPHAVALHD FPA----EQA DDLSLTSGEI VYLLEKI--- --DAEWYRGK CR------NQ TGVFP---AN YVKVIV
P79956/1-59       EEVIVVAKFD YVA----QQD QELDIKKNER LWLLDD---- --SKSWWRVR NS-----TNK TGFVP---SN YVERKN
Q16291/1-60       DKHFVVALYD YTA----MND RDLQMLKGEK LQVLKG---- --TGDWWLAR SLV----TGR EGYVP---SN FVARVE
Q21430/1-59       SPLSARAKFQ FEG----SNN DELSFDKDDI ITITQQQ--- --EGGWWEGT HE------GV TGWFP---CA YVTLIT
BAA34497/1-59     EKLPAKAVYD FKA----QTS KELSFKKGDT VYILRKI--- --DQNWYEGE HH------GR VGIFP---IS YVEKLT
S58653/1-59       SSPKAVALYS FAG----EES GDLPFRKGDV ITILKKSD-- -SQNDWWTGR VN------GR EGIFP---AN YVELV-
SRC1_DROME/1-61   LKRVVVSLYD YKS----RDE SDLSFMKGDR MEVIDDT--- --ESDWWRVV NLT----TRQ EGLIP---LN FVAEER
O08635_1/1-59     KGRKVKALYD FLG----ENE DELSFKAGDV ITELEPI--- --DDAWMRGE LM------GR AGMFP---KN YVQFLQ
O61618_1/1-58     DVEYYIAAYP YES----AEE GDLSFSAGEM VMVIKK---- --EGEWWTGT IG------SR TGMFP---SN YVQKAD
NCK_HUMAN_2/1-5   LNMPAYVKFN YMA----ERE DELSLIKGTK VIVMEKC--- --SDGWWRGS YN------GQ VGWFP---SN YVTEEG
BAA76275/1-60     TPITCKALFD YTA----VRP DELSFCKDAV ITNVEKH--- --GGGWWKGD CG-----NKN QKWFP---AN HVEEVQ
VAV2_HUMAN/1-61   VIGTAVARYN FAA----RDM RELSLREGDV VRIYSRIG-- -GDQGWWKGE TN------GR IGWFP---ST YVEEEG
O61843/1-59       QKPQCRALFD FDA----QSE GELDFKEGTL IELVSQI--- --DENWYEGR VN------GK TGLFP---VT YVQVLV
PIP5_RAT/1-60     PQRTVKALYD YKA----KRS DELTFCRGAL IHNVSKE--- --PGGWWKGD YG-----TRI QQYFP---SN YVEDIS
JN0471/1-60       ELKKVVALYD YMP----MNA NDLQLRKGEE YFILEES--- --NLPWWRAR DK-----NGQ EGYIP---SN YITEAE
O89100/1-59       RVRWARALYD FEA----LEE DELGFRSGEV VEVLDSS--- --NPSWWTGR LH------NK LGLFP---AN YVAPMM
AAD19747_2/1-61   ALVNYRALYP FEA----RNH DEMSFSSGDI IQVDEKTV-- -GEPGWLYGS FQ------GK FGWFP---CN YVEKVL
O43125/1-59       NVERVLAMYD FPG----PDA GDLGFHAGEV IIVLEHV--- --NNDWWRGE LN------GK EGIFP---SN YVRLLE
Q24218_2/1-59     DVCYVVAKYD YAA----QGA QELDLRKNER YLLLDD---- --SKHWWRVQ NS-----RNQ SGYVP---SN YVKKEK
Q16584/1-64       ANPVWTALFD YEP----SGQ DELALRKGDR VEVLSRDAAI SGDEGWWAGQ VG------GQ VGIFP---SN YVSRGG
O43281/1-63       STQLARALYD NTA----ESP QELSFRRGDV LRVLQREGA- GGLDGWCLCS LH------GQ QGIVP---AN RVKLLP
O42455/1-60       SRKVVVALYD YDI----MNP DDLPLRKDQE YVVLEQG--- --AHNWWRAR DA-----QGR EGFIP---SN YVVETR
NCF2_HUMAN/1-59   KGSQVEALFS YEA----TQP EDLEFQEGDI ILVLSKV--- --NEEWLEGE CK------GK VGIFP---KV FVEDCA
S30356/1-64       HLDTVIALYD FPG----TQS SHLPLNLGDT IHVLSKS--- --ATGWWDGV VMGN-SGELQ RGWFP---HN YVRSVN
O00886/1-60       ENLYATALYP YQA----SGQ WHLPFKKDDK IVLLDIK--- -SEEGWLKGE LN------GK IGYFP---AS YVEIIA
SPCA_DROME/1-59   GKECVVALYD YTE----KSP REVSMKKGDV LTLLNSN--- --NKDWWKVE VN------DR QGFVP---AA YIKKID
O08635_2/1-59     PGEWCKALHS FTA----ETS EDLPFKRGDR ILILERL--- --DSDWYRGR LH------DR EGIFP---AV FVQPCP
BAA34489/1-62     SVCFVKALYD YEG----QTD DELSFPEGAI IRILNKEN-- QDDDGFWEGE FN------GR IGVFP---SV LVEELS
A46243/1-60       EAEYVRALFD FNG----NDE EDLPFKKGDI LRIRDKP--- --EEQWWNAE DS-----EGK RGMIP---VP YVEKYR
P78815/1-60       TKTPAIVIYD YSP----EEE NEIELVENEQ IQILEFV--- --DDGWWLGE NS-----KGQ QGLFP---SN YVEITG
P78815_1/1-57     PGKSVKAIYD YQA----QED NELSFFEDEI IANVDCV--- --DPNWWEGE CH------GH RGLFP---SN YVEEI-
AAD16007/1-61     DLCSYQALYS YVP----QND DELELRDGDI VDVMEKC--- --DDGWFVGT SRR----TRQ FGTFP---GN YVKPLY
Q23461/1-60       KFRRVKTLYA CTP----DHH SELSFEPGQI ITNVYES--- -NEDGWLVGT LN------GK TGLIP---SN YVEPLP
BAA34491/1-62     NKGVAYALWD YEA----QNS DELSFHEGDA LTILRRKD-- ESETEWWWAR LG------DR EGYVP---KN LLGLYP
Q14185/1-61       EEKYGVAFYN YDA----RGA DELSLQIGDT VHILET---- --YEGWYRGY TLR---KKSK KGIFP---AS YIHLKE
P97369/1-59       EAPRAEALFD FTG----NSK LELSFKAGDV IFLLSKI--- --NKDWLEGT SQ------GA TGIFP---GS FVKILK
CAA18266/1-59     HRRRAKALLD FER----HDD DELGFRKNDI ITIVSQK--- --DEHCWVGE LN------GL RGWFP---AK FVEVLD
ABL_DROME/1-61    DPQLFVALYD FQA----GGE NQLSLKKGEQ VRILSYN--- -KSGEWCEAH SD-----SGN VGWVP---SN YVTPLN
Q64434/1-64       LGPKYVGLWD FKA----RTD EELSFQAGDL LHVTKK---- --EELWWWAT LLDAEGKALA EGYVP---HN YLAEKE
YBY2_SCHPO/1-63   ILGYVSALYD YDA----AIP EEISFRKGDT IAVLKLY--- --EDGWWEGF VVGE--DDHN RGQFP---SN FVREIE
O70254/1-61       RPSVYVAIYP YTP----RKE DELELRKGEM FLVFERC--- --QDGWYKGT SMH----TSK IGVFP---GN YVAPVT
O13154/1-60       MEVRVRALYD YEG----QEQ DELSFKAGDE LTKMENE--- -DEQGWCKGR LD-----NGQ VGLYP---AN YVEPIQ
O77202/1-56       TVGRCRALYD YGA----QEA DELTLREGDV IDVIQK---- --SGEWWEGT LN------GK TGVFP---AN YVEDI-
Q23507_2/1-59     SEDYVVVKYD YLA----QEE QELTIKKNER LKLLDD---- --SKNWWKVM ND-----SNS VGFVP---SN YVRKES
P70248/1-57       HGPRCRALYQ YIG----QDV DELSFNVNEV IEILIED--- --SSGWWKGR LH------GQ EGLFP---GN YVEKI-
O14365/1-61       ILGYVIALYD YQA----QIP EEISFQKGDT LMVLRTQ--- --EDGWWDGE IINV--PNSK RGLFP---SN FVQTV-
O76133/1-60       FVKLVVALYP FKA----IEG GDLSLEKNAE YEVIDDS--- --QEHWWKVK DA-----LGN VGYIP---SN YVKPKA
```

```
CSK_RAT/1-61       SGTECIAKYN FHG----TAE QDLPFCKGDV LTIVAVT--- -KDPNWYKAK NK-----VGR EGIIP---AN YVQKRE
Q12965/1-57        QVPQCKALYA YDA----QDT DELSFNANDI IDIIKED--- --PSGWWTGR LR------GK PGLFP---NN YVTKI-
G1302660/1-59      GVVEAVACFA YTG----RTA QELSFRRGDV LRLHERA--- --SSDWWRGE HN------GM RGLIP---HK YITLPA
O43586/1-57        PAQEYRALYD YTA----QNP DELDLSAGDI LEVILEG--- --EDGWWTVE RN------GQ RGFVP---GS YLEKL-
BAA36290/1-59      EGIFVEALWD HVT----MDP DELGFKVGDV IRVNDMS--- --NADWWWGE ID------SA EGWFP---AT FVRILV
SS81_YEAST/1-61    FIYKAKALYP YDAD--DDDA YEISFEQNEI LQVSDI---- --EGRWWKAR RA-----NGE TGIIP---SN YVQLID
HS1_HUMAN/1-58     LGISAVALYD YQG----EGS DELSFDPDDV ITDIEMV--- --DEGWWRGR CH------GH FGLFP---AN YVKLLE
AAC98349/1-62      KVRRVKTIYD CQA----DND DELTFIEGEV IIVTGEE--- --DQEWWIGH IEG---QPER KGVFP---VS FVHILS
SLA1_YEAST/1-62    SKKRGIVQYD FMA----ESQ DELTIKSGDK VYILDDK--- -KSKDWWMCQ LVD----SGK SGLVP---AQ FIEPVR
O60504/1-59        KRKAARLKFD FQA----QSP KELTLQKGDI VYIHKEV--- --DKNWLEGE HH------GR LGIFP---AN YVEVLP
O08641/1-59        QPIEVTALYS FEG----QQP GDLNFQAGDR IIVISKTD-- -SNFDWWEGK LR------GQ TGIFP---AN YVTMN-
MYSB_DICDI/1-58    SRPTAKALYD YDA----SST DELSFKEGDI IFIVQKD--- --NGGWTQGE LK-----SGQ KGWAP---TN YLQYN-
TXK_MOUSE/1-60     ERIQVKALYD FLP----REP GNLALKRAEE YLILERC--- --DPHWWKAR DR-----FGN EGLIP---SN YVTENR
O35413_1/1-61      EIGEAIAKYN FNA----DTN VELSLRKGDR IILLKRV--- --DQNWYEGK IPG----TNR QGIFP---VS YVEVVK
TEC_HUMAN/1-60     SEEIVVAMYD FQA----AEG HDLRLERGQE YLILEKN--- --DVHWWRAR DK-----YGN EGYIP---SN YVTGKK
CAA21577/1-60      RVKVYRALYD FQA----RSA QELTFSEGDL MYVSDEQ--- -PNKDWFQAS IG------GK KGLVP---AN YGEYLQ
O62482/1-59        PVMTATAVYK FEP----RSA RELPLNRGDI IRIIREV--- --DGYWMEGE RN------GR SGIFP---TS YVQINT
Q12163/1-61        VNQRAVALYD FEP----END NELRLAEGDI VFISYKH--- --GQGWLVAE NES----GSK TGLVP---EE FVSYIQ
PEXD_PICPA/1-67    KLEFARALYD FNP---ENEE MELKLARGEL MAILSKT(4) NQESTWWKCR SR-----DGK VGFVP---YN YVEIIE
O77132/1-62        NGTECVARYD FKG----NSE KDLPFKKGDI IEILQST--- -RDPNWYNAK KVS----DGR TGLIP---IN YVQQRR
Q13869/1-60        DPNLFVALYD FVA----SGD NTLSITKGEK LRVLGYN--- -HNGEWCEAQ TK------NG QGWVP---SN YITPVN
O15034/1-67        STRRMVALYD YDP(6)VDVE AELTFCTGDI ITVFGEI--- -DEDGFYYGE LN------GQ KGLVP---SN FLEEVP
PEXD_YEAST/1-66    KLEFARALYD FVP---ENPE MEVALKKGDL MAILSKKDPL GRDSDWWKVR TK-----NGN IGYIP---YN YIEIIK
AAD00898/1-62      PRGTARVMYD FAA---EPGN NELTVNEGEI ITITNPD--- -VGGGWMEGR NI-----KGE RGLVP---TD YVEILP
CAA90691/1-73      SAPLFVALYD FHG----VGE EQLSLRKGDQ VRILGYN--- -KNNEWCEAR LYS(11)LGE IGWVP---SN FIAPYN
Q19901/1-56        LNPVVIAVYP YEA----QDV DELSFEAGAE IELMNKD--- --ASGWWQGK VN------NR VGLFP---GN YVKE--
CAB03115/1-62      NTGKVYAAYG YEA----AFE DELSFDAGDE LTVIEKDK-- -VDKNWWTCE KN-----NGE KGQVP---RT YLALYP
NCF1_HUMAN/1-59    ILQTYRAIAD YEK----TSG SEMALSTGDV VEVVEKS--- --ESGWWFCQ MK------AK RGWIP---AS FLEPLD
AAC78231/1-61      QAQIYVATYA YSS----KQD DELSFVVGDL IELISDV--- --EDGWSKGK LKS----TGA VGMFP---TN FVTLKP
O15259/1-60        TGEEYIAVGD FTA----QQV GDLTFKKGEI LLVIEKK--- --PDGWWIAK DA-----KGN EGLVP---RT YLEPYS
Q10929/1-57        YLEKVRVLYD YDA----AKE DELTLRENAI VYVLKKN--- --DDDWYEGV LD------GV TGLFP---GN YVVPV-
O08635_3/1-59      KGPRCVARFE YIG----DQK DELSFSEGEV IILTEYV--- --NEEWGRGE IR------DR SGIFP---LN FVELVG
O62482_1/1-60      DSQKMRAIYP FTA----RSD TELSLKRGEI ITRRRQI--- --DSNWLEGS NQ-----IGI VGIFP---AS YVEPIE
SPK1_DUGTI/1-61    GSYMVKAKYK YAA----SGD TDISFEEKEI MYVLEQF--- --DEFWLKVV KQK----DNK EGLVP---SN YVSKQD
AAC78611_4/1-64    KPEIAQVIAS YTA----TGP EQLTLAPGQL ILIRKKN--- --PGGWWEGE LQAR-GKKRQ IGWFP---AN YVKLLN
O60301/1-61        AIGTCKALYT FEG----QNE GTISVVEGET LYVIEED--- -KGDGWTRIR RN-----EDE EGYVP---TS YVEVCL
O60504_1/1-61      EYGEAVAQYT FKG----DLE VELSFRKGEH ICLIRKV--- --NENWYEGR ITG----TGR QGIFP---AS YVQVSR
ABP1_SACEX/1-60    EAPWATAEYD YEA----GED NELTFAENDK IINIEFV--- --DDDWWLGE LET----TGQ KGLFP---SN YV-VLG
CRKL_MOUSE/1-60    NLEYVRTLYD FPG----NDA EDLPFKKGEL LVIIEKP--- --EEQWWSAR TK-----DGR VGMIP---VP YVEKLV
MYSC_DICDI/1-59    PPQQYIALYE YDA----MQP DELTFKENDV INLIKKV--- --DADWWQGE LVR----TKQ IGMLP---SN YVQQI-
338438/1-59        GEQRVMALYD FQA----RSP REVTMKKGDV LTLLSSI--- --NKDWWKVE AA------DH QGIVP---AV YVRRLA
PEXD_CAEEL/1-65    PHYTAQALFD FQA----SNE QELSFMNGET LRVAPKEEQ- PRVRGWLLAS VAD----GSR IGLVP---IN YVRIVG
Q22070/1-58        KEVQARALRP YRG----TAD DELSFPANVI ITVLRK---- --EEGLWRGR YG------SL TGWFP---SA HVQEIL
EPS8_MOUSE/1-59    PKKYAKSKYD FVA----RNS SELSVMKDDV LEILDD---- --RRQWWKVR NA-----SGD SGFVP---NN ILDIMR
Q99781/1-59        QPEEFVAIAD YAA----TDE TQLSFLRGEK ILILRQT--- --TADWWWGE RA------GC CGYIP---AN HVGKHV
O75791_1/1-56      --MEAVAKFD FTA----SGE DELSFHTGDV LKILSN---- --QEEWFKAE LG------SQ EGYVP---KN FIDIQF
O75563/1-61        YANFYQGLWD CTG----AFS DELSFKRGDV IYILSKEY-- -NRYGWWVGE MK------GA IGLVP---KA YIMEMY
CAA22642/1-66      ETVFVKAIYS FTG----RNE KELDLHTGDV IQVSEQL--- --GPDWYMGE KVN(4)KLGN SGMFP---VN YCTRIY
O15268/1-61        YASYYQGLWD CHG----DQP DELSFQRGDL IRILSKEY-- -NMYGWWVGE LN------SL VGIVP---KE YLTTAF
CAA19485/1-60      VSPYARAVYD FQG----EFE NELSFSADEI ISLRRRI--- --DAEWLEGS IG-----SAR VGIFP---TS FVQIIV
O14259/1-63        IFAYVRALYA YKA----TLP SEIDLNVDDT LVVLNRQ--- --KDGWWKGL VVSP--TVGR IGLFP---SN YIEELE
BEM1_YEAST/1-60    PEKVIKAKYS YQA----QTS KELSFMEGEF FYVSGD---- --EKDWYKAS NPS----TGK EGVVP---KT YFEVFD
SEM5_CAEEL_1/1-    --MEAVAEHD FQA----GSP DELSFKRGNT LKVLNKD--- -EDPHWYKAE LD------GN EGFIP---SN YIRMTE
YB65_SCHPO/1-58    DSSYVKALYA YTA----QSD MELSIQEGDI IQVTNRN--- -AGNGWSEGI LN------GV TGQFP---AN YVTDV-
O43307/1-59        SIVSAEAVWD HVT----MAN RELAFKAGDV IKVLDAS--- --NKDWWWGQ ID------DE EGWFP---AS FVRLWV
Q00647/1-61        GPKKAKALYD FSS----DNN GMLSISAGQI VEIVSKE--- --GNGWWLCM NLE----TSA QGWTP---EA YLEEQV
O13736/1-60        GQKMGTVLYD FIA----EAA DELTVKANMR VVIVNDT--- -ASSDWWKCS VD------GK EGVVP---SN FIKPDT
O43302/1-59        EEEKYVTVQP YTS----QSK DEIGFEKGVT VEVIRKN--- --LEGWWYIR YL------GK EGWAP---AS YLKKAK
NCF1_MOUSE_1/1-    AGEPYVTIKA YAA----VEE DEMSLSEGEA IEVIHKL--- --LDGWWVVR KG------DI TGYFP---SM YLQKAG
O70254_1/1-59      QLPCAKALYN YEG----KEP GDLKFSKGDT IILRRQV--- --DENWYHGE VS------GV HGFFP---TN FVQIIK
YLZ3_CAEEL/1-61    AGFAVKAIYD YAA----ADK DEISFLEGDI IVNCEKI--- --DDGWMTGT VQR----TLQ WGMLP---AN YVQPHK
YMZ9_YEAST/1-62    KEPMFEAAYD FPG---SGSP SELPLKKGDV IYITREE--- --PSGWSLGK LLD----GSK EGWVP---TA YMKPHS
O75044/1-59        EPIEAIAKFD YVG----RTA RELSFKKGAS LLLYQRA--- --SDDWWEGR HN------GI DGLIP---HQ YIVVQD
SCD2_SCHPO/1-62    PRKVIRALYD YTA----RKA TEVSFAKGDF FHVIGRE--- -NDKAWYEVC NPA----AGT RGFVP---VS HFEEIG
```

```
O89032_1/1-59      ILEQYVVVSN YKK----QEN SELSIQAGEV VDVIEKN--- --ESGWWFVS TS------EE QGWVP---AT YLEAQN
O93411/1-60        EEVVLLALYD YDG----VHP GDLTFRKGDH LLLKKE---- --SGEWWEAC LIS----TGE EGFVP---SN YVAYFN
O88903_1/1-58      --VDYIVEYD YDA----VHD DELTIRVGEI IRNVKKL--- -QEEGWLEGE LN------GR RGMFP---DN FVKEIK
PEXD_HUMAN/1-64    DHVVARAEYD FAA----VSE EEISFRAGDM LNLALKEQQ- PKVRGWLLAS LD-----GQT TGLIP---AN YVKILG
O61618_3/1-61      GFVKYQAVYE FNA----RNA EEITFVPGDI ILVPLEQN-- -AEPGWLAGE IN------GH TGWFP---ES YVEKLE
TIM_HUMAN/1-61     NSPQVQCLRA YKP----REN DELALEKADV VMVTQQS--- --SDGWLEGV RLS----DGE RGWFP---VQ QVEFIS
O74653/1-63        SQRFVIALHS FPG----KSS DELPLVEGRK YLLIKMDE-- EFGDGWWEGE DE-----QGN RGIFP---AS HVELIS
BAA34489_1/1-61    YPLTCKVVYS YKA----SQP DELTIEEHEV LEVIEDG--- -DMEDWVKAR NK-----VGQ VGYVP---EK YLQFPT
YE86_SCHPO/1-61    LPCMVRALYA WPG----ERE GDLKFTEGDL IECLSIG--- --DGKWWIGR HIN----TNT QGIFP---SN FVHCLD
AAD04569/1-59      PGRSFMAVKS YQA----QGE GEISLSKGEK IKVLSIG--- --EGGFWEGQ VK------GR VGWFP---SD CLEEVA
Q07533/1-61        PPFKVKARYG WSG----QTK GDLGFLEGDI MEVTRI---- --AGSWFYGK LLR---NKKC SGYFP---HN FVILLE
SLA1_YEAST_1/1-    FLGIYRAVYA YEP----QTP EELAIQEDDL LYLLQKS--- -DIDDWWTVK KRV(4)SEEP VGLVP---ST YIEEAP
YJC0_YEAST/1-64    VPFKVVAQFP YKS----DYE DDLNFEKDQE IIVTSVE--- --DAEWYFGE YQDS-NGDVI EGIFP---KS FVAVQG
O70145_1/1-59      EGEAHRVLFG FVP----ETP EELQVMPGNI VFVLKKG--- --SDNWATVM FN------GQ KGLVP---CN YLEPVE
A53970/1-60        ENVTCKALYS YKA----NKP DELSFPKHAI ITNVQRD--- --NSMWWIGD YG-----GMI KKHLP---AN YVKVID
4454524/1-63       KKQKVKTIFP HTA---GSNK TLLSFAQGDV ITLLIPE--- -EKDGWLYGE HDV----SKA RGWFP---SS YTKLLE
YKA7_CAEEL/1-61    STPYGIAKFD YAP----TQS DEMGLRIGDT VLISKKV--- --DAEWFYGE NQN----QRT FGIVP---SS YLDIKI
O88903_2/1-60      AKEYCRTLFP YTG----TNE DELTFREGEL SLISKET--- -GEAGWWKGE LN------GK EGVFP---DN FAVQIS
O76572/1-61        DCPQALVVHA YQP----SQA DELQLNIGDS LNILRKM--- --PDGWLYGE RAG----DGL GGWFP---SS YVQAII
YKA7_CAEEL_1/1-    SGTYATAIYD YNS----NEA GDLNFAVGSQ IMVTARV--- --NEEWLEGE CF------GR SGIFP---SQ FVDCPN
BOB1_YEAST/1-64    SFPLYIAVNQ YSK----RME DELNMKPGDK IKVITDDG-- EYNDGWYYGR NLR----TKE EGLYP---AV FTKRIA
AAD11957/1-68      PVRIFVALFD YDP(7)DAGE EELPFREGQI LKVFGDK--- -DADGFYQGE GG------GR TGYIP---CN MVAEVA
CC25_YEAST/1-70    PIGIVVAAYD FNY(4)DSSS QLLSVQQGET IYILNKN--- --SSGWWDGL VIDDSNGKVN RGWFP---QN FGRPLR
Q18204/1-60        EICEVQILQS FDG----SAP DDISVKDGES VTVLNIS--- --DPEWTYIR NS-----DNQ SGFVP---SS HVKIPH
VAV2_HUMAN_1/1-    PGPKMVAVQN YHGNPAPPGK PVLTFQTGDV LELLRGD--- -PESPWWEGR LVQ----TRK SGYFP---SS SVKPCP
LAS1_MOUSE/1-59    GGKRYRAVYD YSA----ADE DEVSFQDGDT IVNVQQI--- --DDGWMYGT VER----TGD TGMLP---AN YVEAI-
CAB04592/1-58      AEQNAMALWD YQA----ADD TEISFDPDDI ITDIDQV--- --DSGWWKGR AP-----SGR VGLFP---AN YVKLI-
P91045/1-61        QNPTHRVQSA FHP----RHP DELLLEIGDA VHVDRTA--- --DDHWSYGT NLR----TGQ SGIFP---AS IVCEID
YHR4_YEAST/1-56    PVRTMEAIYA YEA----QGD DEISIDPGDI ITVIRGD--- -DGSGWTYGE CD------GL KGLFP---TS YCK---
CAB19516/1-63      GRMRVKAIFS HAA---GDNS TLLSFKEGDL ITLLVPE--- -ARDGWHYGE SEK----TKM RGWFP---FS YTRVLD
CAA19486/1-59      ---QVKAEYD FQS---QPNT GELSISAGEV LTVIREN--- -IDGGWIEGR NV-----RGS VGLFP---ES YVTPYQ
Q92608/1-61        KERHGVAIYN FQG----SGA PQLSLQIGDV VRIQET---- --CGDWYRGY LIK---HKML QGIFP---KS FIHIKE
O70254_2/1-63      PPPQCKALYD FEVKDKEADK DCLPFAKDDV LTVIRRV--- --DENWAEGM LA------DK IGIFP---IS YVEFNS
O13736_1/1-76      IIGIYKVLYS YEP(10)ENE REISIVEDEI VCLLEKG--- --EDDWYLVK RNV(4)DDEE IGIVP---SN YITEAE
SP02_RAT/1-70      RSLYVRALFD YDR(4)CLPS QGLSFSYGDI LHVINAS--- --DDEWWQAR LVTPHGESEQ IGVIP---SK KRVEKK
O42854/1-64        FPTRVVALYP YRS----SFS DDLEFDPGQV IDVVSNL--- --DGDWYTGT YVDS-DGNRK IGSFP---KD FTEPAE
O14136/1-66        SLEFCKADYE FMS---RDPG VEMSLKKGDI IAILSKTDTQ GNPCEWWQGR KR-----SGE TGWFP---SN YCSIIS
O17972/1-60        RGNVFVAIDS WDA----EAE GDLELIKGKK YRITQTR--- --SDGWWTAL DE-----YGQ RGLVP---KT YLQHVK
ITK_MOUSE/1-60     EETLVIALYD YQT----NDP QELALRCDEE YYLLDSS--- --EIHWWRVQ DK-----NGH EGYAP---SS YLVEKS
O75962/1-65        GCELTVVIHD FTA----CNS NELTIRRGQT VEVLERPH-- -DKPDWCLVR TTDR--SPAA EGLVP---CG SLCIAH
O75117/1-58        PFRKAKALYA CKA----EHD SELSFTAGTV FDNVHPS--- -QEPGWLEGT LN------GK TGLIP---EN YVEFL-
EM55_HUMAN/1-70    LQMFMRAQFD YDP(6)PCKE AGLKFATGDI IQIINKD--- --DSNWWQGR VEGS--SKES AGLIP---SP ELQEWR
Q18165/1-71        KSEYVRALFD YDP(5)VAPH RSMGFNYGDI LHIINSS--- --DDEWWTAR KVHENGEETA EGVIP---SK KRVEKR
Q99140/1-57        MLPHVKSLFP FEG------- QGMKMDKGEV MLLKSKT--- --NDDWWCVR KD-----NGV EGFVP---AN YVREVE
AAC52113/1-70      RGFYIRALFD YDK(4)GFLS QALSFRFGDV LHVIDAS--- --DEEWWQAR RVHSDSETDD IGFIP---SK RRVERR
Q24210/1-69        CEIFVRAQFD YNP(6)PCAQ AGISFQVGDI LQIISKD--- --DHHWWQAR LGT---VGGS AGLIP---SP ELQEWR
SLA1_YEAST_2/1-    --KKVRAIYD YEQV--QNAD EELTFHENDV FDVFDDK--- --DADWLLVK STV----SNE FGFIP---GN YVEPEN
GTPA_HUMAN/1-62    DRRRVRAILP YTK---VPDT DEISFLKGDM FIVHNEL--- --EDGWMWVT NLR----TDE QGLIV---ED LVEEVG
A38747/1-76        EGYQYRALYD YKK----ERE EDIDLHLGDI LTVNKGS(14 )EDIGWLNGY NET----TGE RGDFP---GT YVEYIG
STE6_SCHPO/1-60    MRFQTTAISD YEN---SSNP SFLKFSAGDT IIVIEVL--- --EDGWCDGI CS------EK RGWFP---TS CIDSSK
O70254_3/1-59      VCERHRVVVS YPP----QSE AELELKEGDI VFVHKKR--- --EDGWFKGT LQR----NGK TGLFP---GS FVENI-
AAC78231_1/1-62    TKEMARVKFV YNP----QHD DELALKEIDM LINITNKNC- -GDAGWFEGE LH------GK KGLFP---DN FVELVQ
O62482_2/1-59      GSEMYRAVYP YQP----QKE DELQLYTNDI IFVVEKC--- --DDGWFIGT SLR----TGD FGIFP---GN YVKRH-
Q19253/1-62        VLGEAIAQFA FDG----AQD GTIRMEANEK LWLIEKD--- -EGDGWTRVR KEN----NSA DGFVP---SS YLKVTW
Q99434/1-60        DLPQVEITKA FFA----KQA DEVTLQQADV VLVLQQ---- --EDGWLYGE RLR----DGE TGWFP---ED FARFIT
O00499/1-73        FMFKVQAQHD YTA----TDT DELQLKAGDV VLVIPFQNPE EQDEGWLMGV KES(7)LEKC RGVFP---EN FTERVP
Q17549/1-72        HVQHLRALFD YDP(6)PCKE LAMKFQRGDI LHVLNTK--- --DDNWWQAY RDGEDIQHSL AGLIP---SS SFRQQV
O01761/1-64        SYPVYIAIQD YTPD--KEDV EAIPLEQGQI VEVLDKK--- -NSVRWLVRT KAR----PPR SGWVP---GS YFETPT
YHR4_YEAST_1/1-    DDGKNKVLYA YVQ----KDD DEITITPGDK ISLVARD--- -TGSGWTKIN NDT----TGE TGLVP---TT YIRISS
O54967/1-75        DRPTFVALRD FLL(13)EEP DKLHIQMNDV ITVIEGR--- -AENYWWRGQ NTR----TLC VGPFP---RN VVTSVA
VAV_HUMAN_1/1-6    GLPKMEVFQE YYG(5)GAIG PFLRLNPGDI VELTKAE--- -AEQNWWEGR NTS----TNE IGWFP---CN RVKPYV
O42200/1-60        EERIVVALRN FIP----KEH TDLPLQKDEE YFIVDCS--- --EPNWWTVK DK-----DGN VGSVP---CI YVTEKL
SRM_MOUSE/1-61     RARLFRALYD FTA----RCA EELSVSRGDR LYALKEE--- --GDYIFAQR LSG----PPS TGLVP---VT YLAKAT
MPP2_HUMAN/1-68    RQVFVKCHFD YDP(6)PCKE AGLRFNAGDL LQIVNQD--- --DANWWQAC HVE----GGS AGLIP---SQ LLEEKR
```

```
Q62418/1-59       QGLCARALYD YQA----ADD TEISFDPENL ITGIEVI--- --DEGWWRGY GP-----DGH FGMFP---AN YVELIE
SC25_YEAST/1-72   PIDVVECTYQ YFT----KSR NKLSLRVGDL IYVLTKG--- --SNGWWDGV LIR(11)ILD RGWFP---PS FTRSIL
O02425/1-57       NVPQVEMSYN YEG------- NGMRVSKGEV LALLEKS--- --TPEWWRAL KR-----DGT EGYVP---AN YCKIVP
P97531/1-61       PIGQCVAIYH FEG----SSE GTVSMSEGED LSLMEED--- -KGDGWTRVR RK-----QGG EGYVP---TS YLRVTL
P85B_HUMAN/1-76   EGFQYRALYP FRR----ERP EDLELLPGDV LVVSRAA(14 )QSVGWMPGL NER----TRQ RGDFP---GT YVEFLG
O15034_2/1-67     KVHLCVARYS YNP(5)ENPE AELPLTAGKY LYVYGDM--- -DEDGFYEGE LL-----DGQ RGLVP---SN FVDFVQ
Q60747/1-63       PRTILRKLLQ YMIIYQKTRM MSCPLKEGAI IYVIKKN--- --DDGWFEGV CN------RV TGLFP---GN YVESIM
E305319/1-70      PISMAVALQD YMA----PDC RFLTIHRGQV VYVFSKLK-- GRGRLFWGGS VQG(5)LAAR LGYFP---SS IVREDQ
O60533/1-59       VLYQVVAQHR YSA----QGP EDLGFRQGDT VDVLCEV--- --DQAWLEGH CD------GR IGIFP---KC FVVPAG
YMS2_YEAST/1-68   VIEYAKAMYP LIG---NEAP GLANFHKGDY LLITEIV--- --NKDWYKGE VYD(5)RNHR IGLIP---YN FIQLLH
O89032_2/1-59     QGATYVTCSA YQK----VQD SEISFPEGAE VHVLEKA--- --VSGWWYVR FG------EL EGWAP---SH YLVAEE
O17758/1-66       GFYYAIVQYT FEDPLPSCDA PRLELFIGDR ICVYGQ---- --HGEWGYGR KFDE--KNGK CGIFP---LA YVQIVQ
YB65_SCHPO_1/1-   SKVDGVVLYD FTG----EHE GVITASEGQE FTLLEPD--- -DGSGWVRVK ID------GT DGLIP---AS YVKLND
O88979/1-61       HEQTHRAIFR FVP----RHE DELELEVDDP LLVELQA--- --EDYWYEAY NMR----TGA RGVFP---AY YAIEVT
O88910/1-70       SKVFMRALFH YDP(6)PCQE AGLPFQRRQV LEVVSQD--- --DPTWWQAK RVGD--TNLR AGLIP---SK QFQERR
CAA19485_1/1-61   GIGFANVRHA FSG----RQG DELTVNAGDT VRVLRMV--- --NDEWVMCK DPD----TEK TGIVP---VG FLEVYL
Q13402/1-69       RSKYVVALQD NPNP-AGEES GFLSFAKGDL IILDHDTGEQ VMNSGWANGI NER----TKQ RGDFP---TD CVYVMP
O89032_3/1-59     VEVEYYTIAE FQS----CIS DGISFRGGQK AEVIDKN--- --SGGWWYVQ IG------EK EGWAP---AS YIDKRK
BAA25509/1-68     DSFYIRALYD RLA----DVE QELSFKKDDI LYVDDTLPQ- GTFGSWMAWQ LDEN-AQKIQ RGQIP---SK YVMDQE
YKA7_CAEEL_2/1-   PKQTVTVSYD YDS----GVA SDLRLFEGDV ITALEDI--- --DAQWLLAE CR------GQ QGMVP---KT FLGPPY
Q99771/1-61       REQTHRAVFR FIP----RHP DELELDVDDP VLVEAEE--- --DDFWFRGF NMR----TGE RGVFP---AF YAHAVP
AMPH_HUMAN/1-73   FLYKVETLHD FEA----ANS DELTLQRGDV VLVVPSDSEA DQDAGWLVGV KES(7)LATY KGLFP---EN FTRRLD
CAA19497/1-64     VLCQVKVDYD FLP----QGS NQVEVKEGEV IGVLQRTDD- DGNPEWLLIK RD-----SGK VGYVP---AA YYYVIA
O18249/1-59       GGKLAVVTYD RGG----QNT KELTVHKGEY LEVIFD---- --ERNWWECK NM-----HQR VGYVP---HT ILSMVP
CRKL_MOUSE_1/1-   GPVFAKAIQK RVPC--AYDK TALALEVGDI VKVTRMN--- --INGQWEGE VN------GR KGLFP---FT HVKIFD
Q21004/1-63       TLYFVTSTHN YKA----VDT DELSFEQGVQ IKVIEANEDD QLDDGWRLGE LE-----DGK RGVFP---EN FTKKV-
MYSH_ACACA/1-58   EYKQVEVVYD YDG---GGDA QRLVLVKGAI ITVIKE---- --YEGWAYGS TD-----DGQ VGLYP---IN YTRPI-
SCD2_SCHPO_1/1-   QPLFGIVQFD FAA----ERP DELEAKAGEA IIIIARS--- --NHEWLVAK PIG---RLGG PGLIP---LS FIQLRD
O00235/1-61       NAHNQIAIYA HQP----RTA DEIPMEPGDI IGVAGNH--- --WDGYSKGV NRK----LGR TGLYP---SY KVREKI
Q16176/1-62       PGTQCITKCE HTR----PKP GELAFRKGDV VTILEAC--- -ENKSWYRVK HHT----SGQ EGLLA---AG ALRERE
CAA10073/1-62     DKKRVVAILP YTK---MPET DELSFQKGDI FFVHNDM--- --GDGWLWVT AHR----TGE QGMIF---RE LVDDLD
O16103/1-62       QAEFGIAKCN FDQ---ESKP HRLNLDVGDA VIILKE---- --TTHWYYGY RQK---AKEI RGIFP---KS YIHLCE
AAC79695_1/1-68   GLPKMQVIRN YSG(5)LHEG PPLQLQAGDT VELLKGD--- -AHSLFWQGR NLA----SGE VGFFP---SD AVKPCP
AAD19964/1-65     DSFFIRSHFE CEK----ETP QSLAFTRGEV FRVVDTLYD- GKLGHWLAVR IGN----DLE KGLIP---NK SRAEQM
Q24316/1-61       KPEQLKAVVN CTE----PKK DHLLYRQGDI ISVLDRN--- -TGTPFWKGV LS-----TGK TGYFS---TP FWKGVL
P91182/1-68       PANFHRVQDT YSS(6)PCLW LDLEVNPGDV IQILSK---- --DEKWMQVR KLG---DLTQ VGYLP---TS LTMEKV
O75995/1-61       FCGRARVHTD FTPS--PYDH DSLKLQKGDV IQIIEKP--- -PVGTWLGLL N-------GK VGSFK---FI YVDVLP
AAC72274/1-74     DSFYIRTHFE LEP----SPP SGLGFTRGDV FHVLDTL(8) ARGGHWLAVR MGRD-LREQE RGIIP---NQ SRAEQL
912989/1-65       PKKLVPGKYT VVMDDEKGGP DTLAMRSGDM VEVVEEG--- -AEGLWYVRD LT-----SSK EGWVP---AS SLSTLL
CAB04595/1-63     TAVTVTALFE FAK----SSA ETMSIEQGEI LLVLEHD--- -HGDGWTRTK NCRK--HNEE SGFVP---TS YLQFPQ
YAI4_YEAST/1-61   DPDKLYALYA FNG----HDS SHCQLGQDEP CILLNDQ--- --DAYWWLVK RIT----DGK IGFAP---AE ILETFP
A57152/1-60       ESDFLAVLTD YPS----SDI SPPIFRRGEK LRVISD---- --EGGWWKAI SLS----TGR ESYIP---GI CVARVY
171531/1-76       LGKTYTVIQD YEP----RLT DEIRISLGEK VKILATH--- --TDGWCLVE KCN(16)NED RGIVP---GD CLQEYD
O45232/1-65       RAQSVRAVVD CKD----SAA DHLHFKKDDL IVVISRSPAQ YPDGYYWFGS LR-----NGK LGLFR---PT DTVAHL
YWR1_CAEEL/1-60   MFLDAVARET YNS----IQP GALQLTKGDE VVVVENT--- --GQDWFGQN KK-----NQK FGTFP---RS VVFAQT
AAD15528/1-62     GTRFGTALYD FTA----GGD DELNLTAEEE LEIEYE---- --VDGWFYVK KKRPGRDGKM AGLVP---VL YVNQS-
O61528/1-65       SSEVWIVTAD FDG----QVE GHLTVHKGDR VEIVEDQA-- TDCAEYVQVV LCD---QPTK HGLVP---AS IIAPPE
O16882/1-61       QAHEVIVIED HIA----QNN KEIDLKVGDK VGIAGNH--- --WNGYSKGT NRQ----TYK EGVFP---SY KIRIYF
Q20622/1-58       YGSILIVNDD FNA----SSG EQMTVNRGDK VILLKCG--- --SRGWVFVR DSI----SNR TGWVP---EP YVNP--
O60132/1-61       DCNFVHAIRG FEA----TVE GQVDATKGDM MILLDDS--- --NSYWWLVK MCK----NLA IGYLP---AE YIETPS
O43302_4/1-61     LKDVYVSIAD YEG-----DE ETAGFQEGVS MEVLERN--- --PNGWWYCQ ILDG--VKPF KGWVP---SN YLEKKN
Q94880/1-68       DSFHIKTHFH CDN----PSK GEMAFKAGDV FRVIDTLHN- GVVGSWQVLK IGRG-HQEMQ RGVIP---NK SRAEEL
O13736_2/1-58     S-TKMKALYD YTQ----QSV DEISFQADQT LDCYGDT--- -DSDWILVGF N-------NN FGLAP---RN YVEGMD
O00359/1-61       VIHLAKACCD VKG-----GK NELSFKQGEQ IEIIRITD-- -NPEGKWLGR TA-----RGS YGYIK---TT AVEIDY
O44222/1-69       VAFAMRANYG YNG(6)PIHG MALSFEPKDF LHIKEKF--- --NNDWLIGR VVR---EGCD IGFIP---SP SKLESL
O45009/1-74       VAFAVQAMRD YDG(6)PLKD HTISFNTNDF IHIHTKF--- --NSDWWIGR IVR---SHSE FGFVP(5)AN YMVSDE
CAB38822/1-59     GYFLGEVMFP YQA----DSD FELSLSVGDY VVIREVV--- --SSVWAEGE CK------GN AGWFT---YI YIERRD
CCB4_HUMAN/1-69   VAFAVKTNVS YCG(6)PVPS TAISFDAKDF LHIKEKY--- --NNDWWIGR LVK---EGCE IGFIP---SP LRLENI
Q18204_1/1-59     PYTPGQLTKN FTA----TVA DEISVTRGTT VKAIYR---- --DDQWIYVE VS-----DGR KGFVP---QT YCKLLL
O70395/1-86       DSDYVVAVRN FLS----EDP ELLSFHKGDI IHLQSLE(26 )PDFGWRFGA VH------GR VGRFP---SE LVQPAA
Q13470/1-101      EAGPSEACCV RDA----TEP GALRMETGDP ITVIEGS(4) SPDSTIWKDQ NGR----TFK VGSFP---AS AVTLTD
A54844/1-68       VAFAVRTNVA YDA(6)PVQG GAVSFDVREF LHIKEKY--- --DHNWWIGR LVK---EGCD VGFIP---SP -VKLDH
BEM1_YEAST_1/1-   GSLYAIVLYD FKA----EKA DELTTYVGEN LFICAHH--- --NCEWFIAK PIG---RLGG PGLVP---VG FVSIID
Q18204_2/1-61     ENENLLVVEP FSG----RSP LDLTVRPGEW IKCTGRP--- --VDDWLWAV RIA----DEK QGFIP---NK VVILAT
```

```
Q20005/1-59       GTKQAKVIMD YDA----VLP QEISVTQNDI LIVYRLPG-- -MDAEYVMAE KG------GK RGKLP---VS YIELL-
O77291/1-58       RQGPVQAICA YKQ------Q GQLQIEKGET VTLLDNS--- -GRVKWRVRT AK------GQ EGPIP---GA CLLLPP
B0303.7_3/1-62    SPKKAVATGD YHS----EDP KHLYVTRGDH LLIVEDV--- --DDYYYKGK LEAF--KTLP AGILP---KN IVKLEN
4521278/1-65      GTSSMAVIKD YYA----LKE NEICVSQGEV VQVLAVN--- --QQNMCLVY QPASDHSPAA EGWVP---GS ILAPLT
O08635_4/1-59     SVPHGIANED IVS----RNP TELSCKRGDV LVILKQA--- --ENNYLECQ RG------EG TGRVH---PS QMKIVT
Consensus/60%     ...bsbAlac apt....pss s-LsbppG-b lpllpc.... ..sssWaptc h.......tp pGbbP...ss YVpbb.
```

Send comments to Ivica Letunic
