# Supplementary material for: Insights into Protein Aggregation by NMR Characterization of Insoluble SH3 Mutants Solubilized in Salt-Free Water
Source: PLoS One. 2009 Nov 23;4(11):e7805. doi: 10.1371/journal.pone.0007805 (PMC2776303; doi:10.1371/journal.pone.0007805)
Supplement: Table S1 — Native-like long-range NOEs persistent in V22-SH3 (0.03 MB DOC) [file pone.0007805.s003.doc]

| **Secondary Structure**  **Regions** | **NOEs** |
| --- | --- |
| **Between the first and second beta strands** | Val3 HN Leu26 HB  Val3 HA Trp27 HE |
| **Between the second and third beta strands** | Arg25 HN Asn40 HB  Trp27 HE Asn40 HD  Trp27 HE Asn40 HB  Trp27 HE Arg39 HB  Leu29 HN Arg39 HE  Trp35 HE Asp31 HA |
| **Between the third and fourth beta strands** | Arg39 HN Thr45 HA  Asn40 HN Thr45 HA  Arg44 HH Arg39 HB  Tyr47 HN Arg37 HA  Tyr47 HN Trp36 HB  Tyr47 HN Val38 HB  Val48 HN Trp35 HA |
| **Between the RT loop and fourth beta strand** | Leu17 HN Thr45 HA  Arg44 HH Leu17 HD |
| **Between the two RT loop strands** | Lys20 HN Trp7 HA  Lys20 HN Trp7 HB  Trp7 HE Lys20 HG  Tyr9 HN Ile19 HA  Tyr9 HN Asp18 HB  Tyr9 HN Asp18 HB  Tyr9 HN Ile19 HB |
