# Supplementary material for: Insights into Protein Aggregation by NMR Characterization of Insoluble SH3 Mutants Solubilized in Salt-Free Water
Source: PLoS One. 2009 Nov 23;4(11):e7805. doi: 10.1371/journal.pone.0007805 (PMC2776303; doi:10.1371/journal.pone.0007805)
Supplement: Table S2 — Non-native NOEs identified in V22-SH3 (0.03 MB DOC) [file pone.0007805.s004.doc]

| **The first beta-strand**  Val3 HN Ile4 HN  Ala5 HN Val3 HA  Ala5 HN Val3 HB  **Loop and two RT-loop strands**  Trp7 HN Val3 HB  Trp7 HN Val3 HG  Trp7 HN Ile4 HG  Trp7 HN Ala5 HB  Trp7 HN Ala5 HA  Asp8 HN Ala5 HB  Asp8 HN Lys6 HB  Asp8 HN Lys6 HE  Asp8 HN Ala11 HB  Thr10 HN Trp7 HA  Thr10 HN Trp7 HB  Ala11 HN Trp7 HA  Thr10 HN Gln12 HB  Thr10 HN Gln12 HG  Lys20 HN Val22 HN  Val22 HN Glu24 HA  Val22 HN Lys20 HB  Val22 HN Lys20 HG  Val22 HN Lys20 HA  Val22 HN Lys20 HD  Asn23 HN Arg25 HG  **The second beta-strand**  Leu26 HN Glu24 HB  Leu26 HN Glu24 HA  Leu29 HN Trp27 HA  Asp31 HN Leu29 HA  Asp31 HN Leu29 HB  **The third beta-strand**  Arg37 HN Val38 HN  Arg37 HN Trp36 HN  Asn40 HN Val38 HA  Asn40 HN Val38 HB  Arg37 HN Arg39 HA  **The fourth beta-strand**  Tyr47 HN Gly46 HN  Tyr47 HN Thr45 HG  **The fifth beta-strand**  Tyr52 HN Glu54 HG  Val53 HN Tyr52 HN  Glu54 HN Arg55 HN  Glu54 HN Val53 HN  Glu54 HN Tyr52 HB  Arg55 HN Val53 HG  Arg55 HN Val53 HB |
| --- |
